# A stem-loop RNA RIG-I agonist protects against acute and chronic SARS-CoV-2 infection in mice

**DOI:** 10.1084/jem.20211818

**Published:** 2021-11-10

**Authors:** Tianyang Mao, Benjamin Israelow, Carolina Lucas, Chantal B.F. Vogels, Maria Luisa Gomez-Calvo, Olga Fedorova, Mallery I. Breban, Bridget L. Menasche, Huiping Dong, Melissa Linehan, Yale SARS-CoV-2 Genome Surveillance Initiative, Craig B. Wilen, Marie L. Landry, Nathan D. Grubaugh, Anna M. Pyle, Akiko Iwasaki

**Affiliations:** 1 Department of Immunobiology, Yale School of Medicine, New Haven, CT; 2 Department of Internal Medicine, Section of Infectious Diseases, Yale School of Medicine, New Haven, CT; 3 Department of Epidemiology of Microbial Diseases, Yale School of Public Health, New Haven, CT; 4 Department of Molecular, Cellular and Developmental Biology, Yale University, New Haven, CT; 5 Department of Laboratory Medicine, Yale School of Medicine, New Haven, CT; 6 Department of Ecology and Evolutionary Biology, Yale University, New Haven, CT; 7 Department of Chemistry, Yale University, New Haven, CT; 8 Howard Hughes Medical Institute, Chevy Chase, MD

## Abstract

As SARS-CoV-2 continues to cause morbidity and mortality around the world, there is an urgent need for the development of effective medical countermeasures. Here, we assessed the antiviral capacity of a minimal RIG-I agonist, stem-loop RNA 14 (SLR14), in viral control, disease prevention, post-infection therapy, and cross-variant protection in mouse models of SARS-CoV-2 infection. A single dose of SLR14 prevented viral infection in the lower respiratory tract and development of severe disease in a type I interferon (IFN-I)–dependent manner. SLR14 demonstrated remarkable prophylactic protective capacity against lethal SARS-CoV-2 infection and retained considerable efficacy as a therapeutic agent. In immunodeficient mice carrying chronic SARS-CoV-2 infection, SLR14 elicited near-sterilizing innate immunity in the absence of the adaptive immune system. In the context of infection with variants of concern (VOCs), SLR14 conferred broad protection against emerging VOCs. These findings demonstrate the therapeutic potential of SLR14 as a host-directed, broad-spectrum antiviral for early post-exposure treatment and treatment of chronically infected immunosuppressed patients.

## Teaser

A stem-loop RNA (SLR) RIG-I agonist is effective in preventing and treating acute SARS-CoV-2 infection in mice. A single injection of SLR can clear chronic SARS-CoV-2 in immunocompromised mice. SLR is effective against the ancestral virus as well as variants of concern.

## Graphical Abstract

**Figure:**
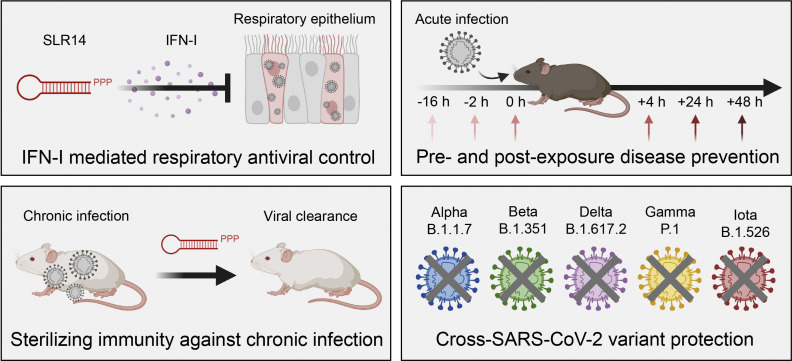

## Introduction

SARS-CoV-2 is an enveloped, positive-strand RNA virus that causes both upper and lower respiratory infection in humans and other animals (V’Kovski et al., 2021). As of October 26, 2021, the ongoing global COVID-19 pandemic caused by SARS-CoV-2 has led to 243.86 million confirmed cases and 4.95 million deaths worldwide, inflicting widespread economic, sociological, and psychological damage. The clinical spectrum of SARS-CoV-2 infection is wide. While most infections are asymptomatic or mild, older patients, particularly those with underlying medical comorbidities and male sex, are more likely to develop severe diseases involving acute respiratory distress syndrome, multiorgan failure, and death (Hu et al., 2021). Currently, there is a paucity of effective antivirals to treat COVID-19, with remdesivir and monoclonal antibodies demonstrating modest efficacy in a select subset of patients (Beigel et al., 2020; Taylor et al., 2021). To halt substantial morbidity and mortality from COVID-19 around the globe, in addition to the use of vaccines in preventing the disease, efforts are required to develop efficacious therapeutics against SARS-CoV-2.

Great strides made in the understanding of COVID-19 immunology have provided crucial insights into the central role of IFN-I in host immune responses against SARS-CoV-2 infection (Lowery et al., 2021; Park and Iwasaki, 2020). The innate immune system utilizes host-encoded nucleic acid sensors, known as the pattern recognition receptors (PRRs), to surveil viral pathogens by detecting their pathogen-associated molecular patterns (Iwasaki and Medzhitov, 2015). Following SARS-CoV-2 infection, multiple cytosolic PRRs, including RIG-I, MDA-5, and LGP2, mediate viral RNA recognition in infected lung epithelial cells and initiate front-line antiviral defense through IFN-I–dependent and independent mechanisms (Yamada et al., 2021; Yin et al., 2021). Upon secretion, IFN-I engages with its universally expressed receptor in autocrine and paracrine fashions, stimulating the expression of a large network of IFN-stimulated genes (ISGs) to inhibit viral replication (Schneider et al., 2014) and cytokines and chemokines to recruit specialized immune cells to sites of infection. In the context of infection with SARS-CoV-2, IFNs appear to play dichotomous roles. While delayed and prolonged IFN-I and type-III IFN (IFN-III) are associated with severe disease, an early, robust, and regulated production of IFN is protective against COVID-19 (Carvalho et al., 2021; Lucas et al., 2020). This is well exemplified by the susceptibility to life-threatening disease of SARS-CoV-2–infected individuals with inborn defects in IFN-I production and signaling or neutralizing autoantibodies against IFN-I (Bastard et al., 2020; Zhang et al., 2020). COVID-19 patients found with anti–IFN-I autoantibodies demonstrate significantly delayed virological clearance relative to patients without such autoantibodies (Wang et al., 2021). In a mouse model of SARS-CoV-2 infection, early IFN-I blockade leads to exacerbation of disease severity (Wang et al., 2021). Collectively, these studies highlight the beneficial role of IFN-I in SARS-CoV-2 infection and suggest innate immune sensors as promising therapeutic targets to be harnessed for prevention and treatment of COVID-19.

The innate immune system can be pharmacologically modulated to elicit tailored effector outputs with desired immunological outcomes (Demaria et al., 2019; Vanpouille-Box et al., 2019). Given the importance of timely induction of IFN-I in SARS-CoV-2 infection, PRRs can be activated in a targeted manner to induce antiviral protection (Goulet et al., 2013). Our approach in leveraging a synthetic activator of antiviral immunity to combat SARS-CoV-2 builds on our previous work demonstrating that short, tri-, or di-phosphorylated stem-loop RNAs (SLRs) act as specific and potent agonists for the cytosolic RNA sensor RIG-I (Linehan et al., 2018; Luo et al., 2011). SLRs are designed to mimic physiological double-stranded RNA ligands for RIG-I by stably folding into a minimal ligand containing 14-bp RNA duplex (hence the name SLR14) and a tri- or di-phosphorylated 5′ terminus. Each SLR14 presents a single duplex terminus and productively binds one RIG-I molecule. The opposite end of the duplex is blocked with a stable RNA tetraloop to ensure that the RIG-I–SLR14 interaction is structurally defined and resistant to nucleases and strand dissociation. Unlike polyinosinic/polycytidylic acid (poly(I:C)), which is a widely used double-stranded RNA ligand of unknown structure recognized by a handful of PRRs, SLR14 specifically activates RIG-I and triggers an IFN-I–dominant innate immune response (over an IFN-III response) characterized by the induction of multiple IFN-I members within 2 h of i.v. injection in mice (Linehan et al., 2018). Growing evidence suggests that recombinant IFN (rIFN)–based intervention during the early stage of COVID-19 could provide desired clinical benefits in humans. However, rIFN therapy is costly (Nguyen et al., 2020) and can be rendered ineffective by the induction of binding and/or neutralizing anti-drug antibodies (Giovannoni et al., 2002; Matsuda et al., 2012). In contrast, SLR14 is highly manufacturable and can elicit a broad and diverse IFN response. Altogether, with its synthetic simplicity, chemically defined composition, targeted receptor binding, breadth of downstream effector responses, and in vivo potency, SLR14 holds great promise as a new class of RNA therapeutics that can be applied as antivirals against SARS-CoV-2.

While most individuals effectively clear SARS-CoV-2 infection, growing evidence suggests that infection in immunocompromised patients, such as those with severe forms of B cell and antibody deficiency, can become chronic (Aydillo et al., 2020; Choi et al., 2020). In these patients, persistent infection can also foster continuous intrahost viral evolution and lead to further emergence of immune-evasive variants, likely as a result of selective pressure driven by insufficient natural or transferred antibodies. While some patient case reports have used convalescent plasma (CP) to treat chronic SARS-CoV-2 infection, currently there are no approved therapeutic options (Carvelli et al., 2020). Although vaccines currently approved against SARS-CoV-2 are effective at preventing severe disease and death in individuals with an intact immune system, their immunogenicity is significantly attenuated in immunocompromised patients, eliciting suboptimal humoral immune responses (Deepak et al., 2021). Therefore, therapeutic strategies that exert strong antiviral effect independent of adaptive immunity in the setting of immunosuppression are in dire need.

Since the initial outbreak, multiple SARS-CoV-2 variants have emerged with increased transmissibility and altered immunogenicity. The B.1.1.7 lineage (Alpha) was first detected in September 2020 and estimated to be more transmissible than other lineages (Alpert et al., 2021; Washington et al., 2021). While mutations accumulated by B.1.1.7 seem to have negligible impact on infection- and vaccine-induced antibody immunity (Collier et al., 2021; Planas et al., 2021a), other variants have been found to acquire mutations on their spike proteins that can evade antibody targeting. Notably, variant B.1.351 (Beta) and P.1 (Gamma) have both demonstrated considerable resistance to antibody binding and neutralization (Hoffmann et al., 2021; Zhou et al., 2021a). In addition, B.1.526 (Iota) has also exhibited some level of antibody evasion (Zhou et al., 2021b *Preprint*). More recently, B.1.617.2 (Delta) emerged with significantly enhanced transmissibility (40% to 60% increase compared with B.1.1.7), a substantially higher viral replication rate, and heightened neutralization resistance to CP, monoclonal antibodies, and sera from vaccinated individuals (Li et al., 2021a *Preprint*; Lucas et al., 2021; Planas et al., 2021b). In addition, these variants harbor mutations outside the spike protein that may enable strong antagonism of the host antiviral innate immunity. In this context, the containment of COVID-19 will require prophylactic and therapeutic antiviral strategies that afford cross-variant protection.

## Results

### A single dose of SLR14 confers potent antiviral protection against lethal SARS-CoV-2 infection

**Figure 1.**
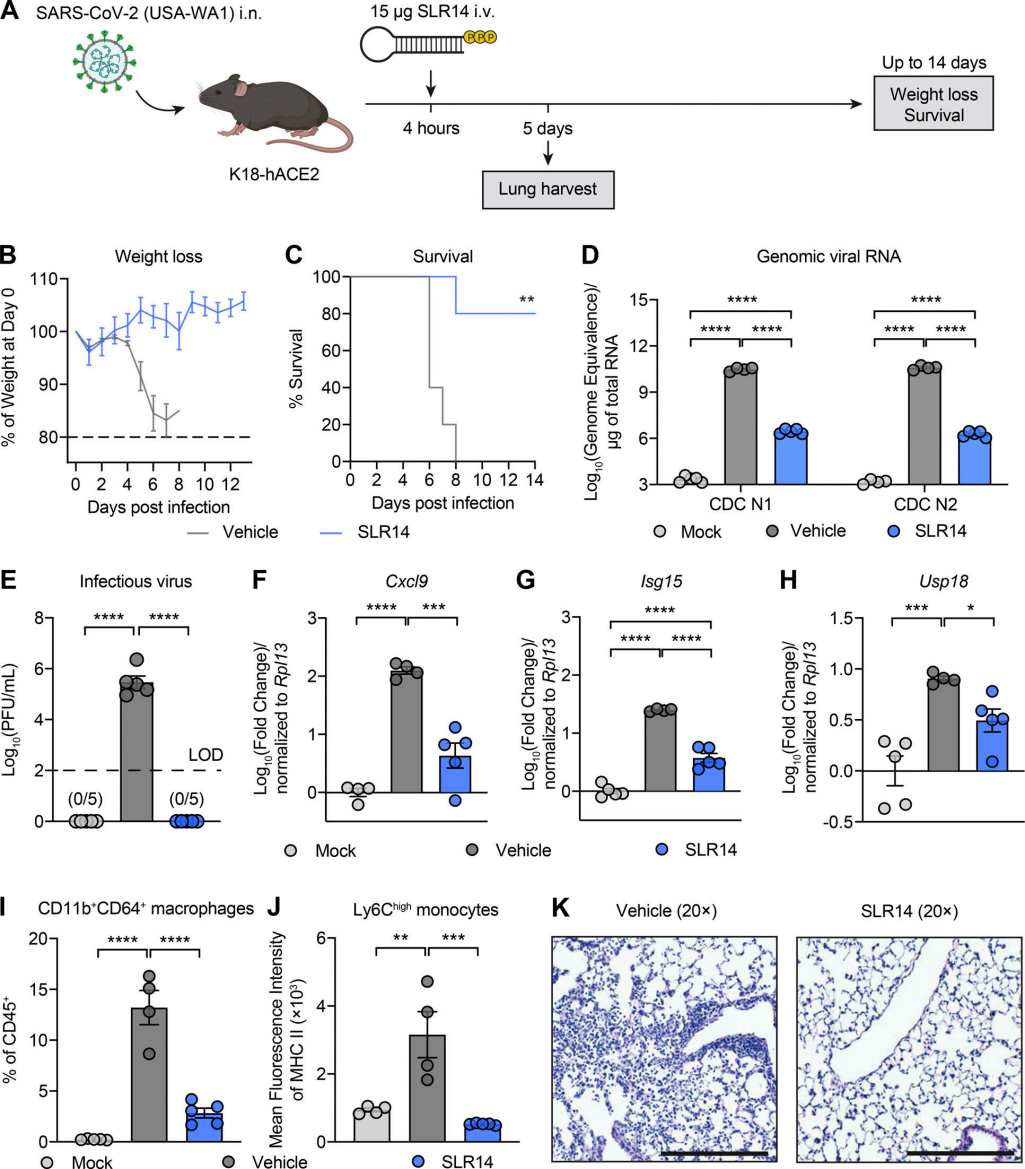
**Single-dose SLR14 induces protective antiviral immunity against SARS-CoV-2 infection. (A)** Experimental scheme. K18-hACE2 mice were intranasally infected with 10^3^ PFU SARS-CoV-2 (2019n-CoV/USA_WA1/2020). 4 h after infection, 15 µg SLR14 or vehicle was i.v. administered. Weight loss and survival were monitored daily up to 14 DPI. Death was recorded when mice were found dead in the cage, moribund, or at 80% of original body weight. In a separate cohort, lung tissues were collected for virological, immunological, and histological analysis 5 DPI. **(B and C)** Weight loss and survival of SLR14- and vehicle-treated K18-hACE2 mice from 1 to 14 DPI. **(D)** Measurement of vRNA in the lung at 5 DPI by RT-qPCR against SARS-CoV-2 N gene using CDCN1 or CDCN2 primer-probe sets. **(E)** Measurement of infectious virus titer in the lung at 5 DPI by plaque assay. Limit of detection (LOD): 10^2^ PFU/ml. **(F–H)** Measurement of expression of the ISGs *Cxcl9*, *Isg15*, and *Usp18* in the lung at 5 DPI by RT-qPCR. **(I and J)** Frequency of CD11b^+^CD64^+^ macrophages of CD45^+^ cells and mean fluorescence intensity of MHC class II on Ly6C^high^ monocytes in the lung at 5 DPI by flow cytometry. **(K)** H&E staining of lung sections from vehicle-treated (left) or SLR14-treated (right) K18-hACE2 mice at 5 DPI. Mean ± SEM; statistical significance was calculated by log-rank Mantel–Cox test (C) or one-way ANOVA followed by Tukey correction (D–J). Scale bars, 250 µm. *, P ≤ 0.05; **, P ≤ 0.01; ***, P ≤ 0.001; ****, P ≤ 0.0001. Data are representative of two independent experiments. Images are representative of *n* = 5 per group.

To examine the antiviral activity of SLR14 in vivo, we used a mouse model of SARS-CoV-2 infection that transgenically expresses human angiotensin-converting enzyme 2 (ACE2) under the keratin 18 gene promoter, also known as the K18-hACE2 mice (McCray et al., 2007). Intranasal infection with SARS-CoV-2 in K18-hACE2 mice leads to viral replication, pulmonary inflammation, and respiratory dysfunction, recapitulating key aspects of infection and pathogenesis seen in patients with COVID-19 (Winkler et al., 2020; Zheng et al., 2021). We have previously shown that i.v. injection of SLR14 complexed with polyethyleneimine results in a rapid, short-lived, and systemic IFN-I response that peaks as early as 2 h after injection and declines to undetectable levels within 24 h of injection (Linehan et al., 2018). Based on this, we intranasally infected K18-hACE2 mice with the ancestral strain of SARS-CoV-2 (2019n-CoV/USA_WA1/2020), administered SLR14 i.v. 4 h after infection, and monitored survival and weight loss daily thereafter (Fig. 1 A). SLR14 treatment considerably prevented weight loss and dramatically improved survival following the infection (Fig. 1, B and C). In contrast, vehicle-treated mice uniformly lost weight and developed apparent signs of sickness behaviors such as reduced motility and hyporesponsiveness, rapidly succumbing to infection by 8 d post-infection (DPI). These results showed that SLR14 effectively alleviates morbidity and reduces mortality, affording protection against lethal SARS-CoV-2 infection in vivo.

To investigate the mechanisms by which SLR14 mediates protection, we collected lung tissues from naive as well as infected mice treated with SLR14 or vehicle 5 DPI. Given the crucial role for RIG-I activation in the initiation of antiviral immunity, we first assessed the impact of SLR14 treatment on lung viral burden. We observed striking reduction in the level of viral genomic RNA (vRNA; by RT-qPCR) and complete clearance of infectious virus (by plaque assay) in lung tissues from SLR14-treated mice compared with vehicle control (Fig. 1, D and E). These results confirm that SLR14 affords protection against SARS-CoV-2 by efficiently mediating viral clearance in the lung tissue. Consistent with the absence of infectious virus, we found significantly attenuated expression of ISGs, including *Cxcl9*, *Isg15*, and *Usp18*, in lung tissues from SLR14-treated mice at this time point (Fig. 1, F–H). In contrast, abundant ISG expression was detected in lungs from vehicle-treated mice, likely resulting from high viral burden.

**Figure S1.**
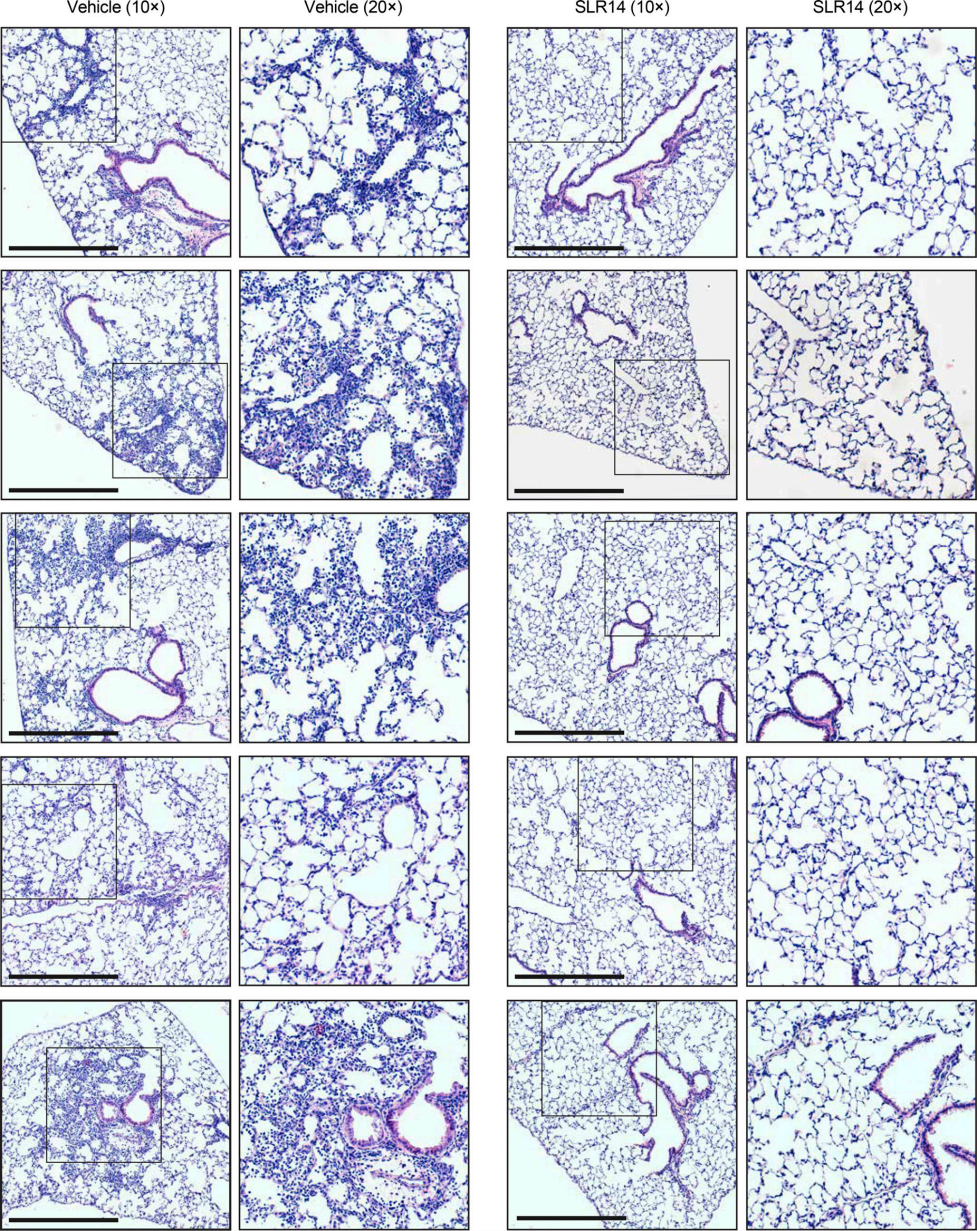
**SLR14 protects against infection-induced viral pneumonia.** H&E staining of lung sections from vehicle- (left) or SLR14-treated (right) K18-hACE2 mice 5 DPI. Images show low- or high-power magnification. Images are representative of *n* = 5 per group. Scale bars, 500 µm.

To further probe the impact of SLR14 on lung immunopathology, we assessed lung immune infiltrates by flow cytometry. We observed markedly decreased CD11b^+^Ly6C^+^ monocyte-derived proinflammatory macrophages in SLR14-treated mice 5 DPI (Fig. 1 I). Additionally, SLR14 treatment led to a significant reduction in the surface expression of MHC class II molecules on Ly6C^high^ monocytes (Fig. 1 J). To directly assess the impact of SLR14 on immunopathology, we performed histological analyses on H&E-stained lung sections from SARS-CoV-2–infected K18-hACE2 mice treated with vehicle or SLR14 5 DPI. Consistent with previous studies (Winkler et al., 2020), we found widespread viral pneumonia associated with immune infiltration at alveolar and interstitial locations in lung sections from SARS-CoV-2–infected vehicle-treated mice (Fig. 1 K and Fig. S1). In contrast, we found minimal inflammatory infiltrates in lung tissues from SLR14-treated mice. Together, these results indicated that in addition to providing viral control, SLR14 protects lung tissues from SARS-CoV-2 infection–induced viral pneumonia.

### SLR14-mediated protection against SARS-CoV-2 depends on IFN-I signaling

**Figure S2.**
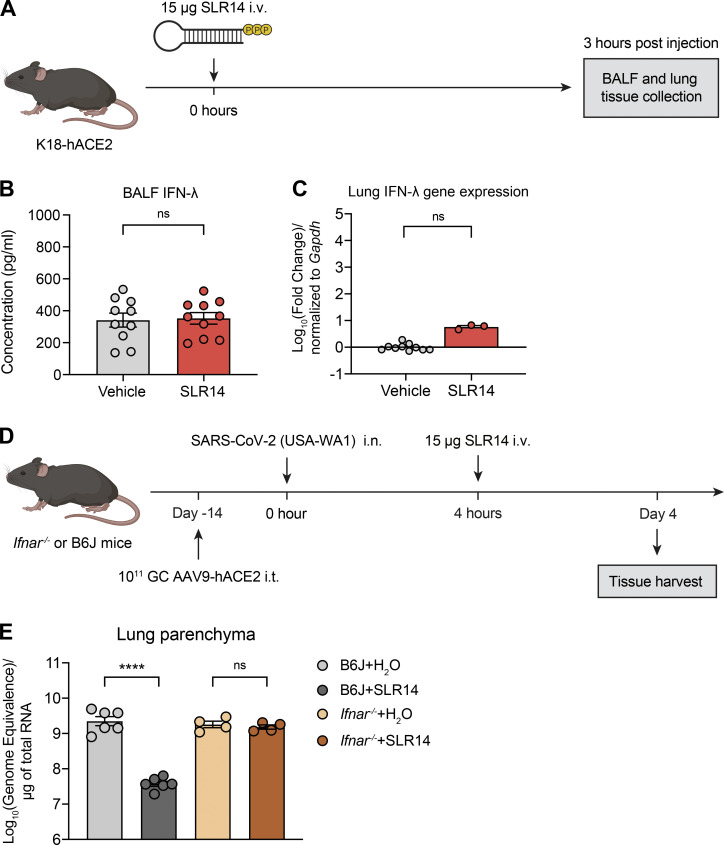
**SLR14 does not significantly elicit IFN-III responses in the respiratory tract. (A–C)** Experimental scheme. K18-hACE2 mice were i.v. administered with 15 µg SLR14 or vehicle. 3 h after injection, BALF and lung tissues were collected for IFN-λ ELISA (B) and RT-qPCR (C), respectively. **(D and E)** Experimental scheme. *Ifnar*^−/−^ mice were intratracheally administered with 10^11^ genome copies of AAV9-hACE2 and allowed to rest for 2 wk before intranasal infection with 10^6^ PFU SARS-CoV-2 (2019n-CoV/USA_WA1/2020). 15 µg SLR14 or vehicle were i.v. administered at 4 h after infection. Lung tissues were collected for virological analysis at 4 DPI. Measurement of vRNA at 4 DPI by RT-qPCR using the CDCN2 primer-probe set (E). Mean ± SEM; statistical significance was calculated by two-way ANOVA followed by Bonferroni correction (B and C) or one-way ANOVA followed by Tukey correction (E); ****, P ≤ 0.0001. Data are representative of two independent experiments.

**Figure 2.**
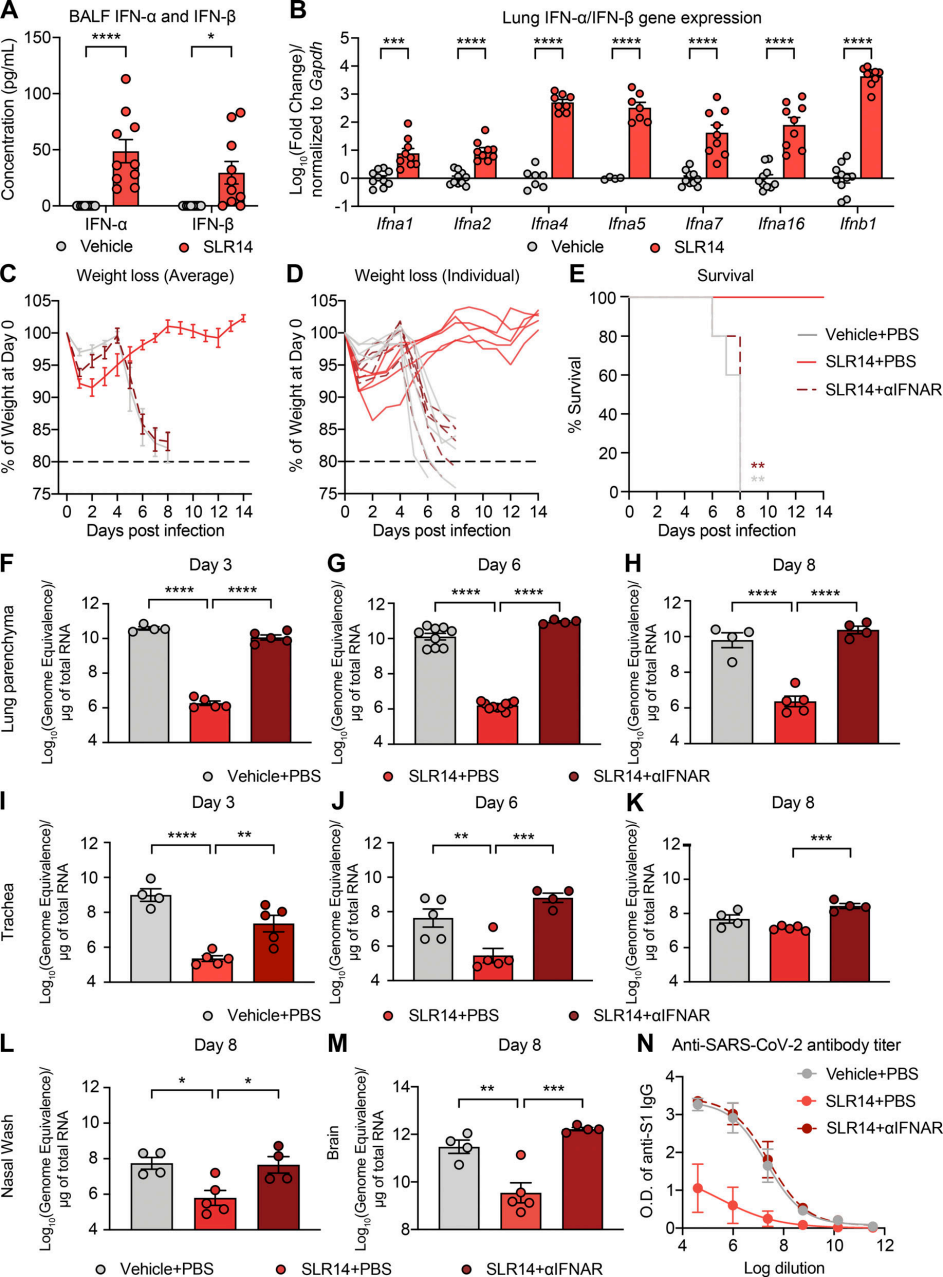
**SLR14-mediated disease prevention and antiviral control rely on IFN-I signaling. (A and B)** Experimental scheme. K18-hACE2 mice were i.v. administered with 15 µg SLR14 or vehicle. 3 h after injection, BALF and lung tissues were collected for IFN-I ELISA (A) and RT-qPCR (B), respectively. **(C–M)** Experimental scheme. K18-hACE2 mice were intranasally infected with 10^3^ PFU SARS-CoV-2 (2019n-CoV/USA_WA1/2020). 2 h before infection, 15 µg SLR14 or vehicle was i.v. administered. 24 h before SLR14 injection, half of the SLR14-treated mice were additionally given 2 mg anti-IFNAR antibodies. Weight loss and survival were monitored daily up to 14 DPI. In a separate cohort, lung and trachea tissues were collected for virological analysis 3, 6, and 8 DPI. Nasal washes and brain tissues were collected for virological analysis at 8 DPI. **(C–E)** Weight loss and survival of K18-hACE2 mice treated with vehicle + PBS, SLR14 + PBS, or SLR14 + αIFNAR from 1 to 14 DPI. **(F–H)** Measurement of vRNA in the lung parenchyma 3, 6, and 8 DPI by RT-qPCR using the CDCN2 primer-probe set. **(I–K)** Measurement of vRNA in the trachea 3, 6, and 8 DPI by RT-qPCR using the CDCN2 primer-probe set. **(L and M)** Measurement of vRNA in the nasal wash (L) or the brain (M) 8 DPI by RT-qPCR using the CDCN2 primer-probe set. **(N)** The experimental scheme was similar to that of Fig. 2, C–M, with the exception that mice were infected with a sublethal dose of SARS-CoV-2. Sera were then collected from survivor mice 14 DPI and used for anti–SARS-CoV-2 S1 IgG measurement by ELISA. Mean ± SEM; statistical significance was calculated by two-way ANOVA followed by Bonferroni correction (A and B), log-rank Mantel–Cox test (E), or one-way ANOVA followed by Tukey correction (F–M); *, P ≤ 0.05; **, P ≤ 0.01; ***, P ≤ 0.001; ****, P ≤ 0.0001. Data are pooled from or representative of two independent experiments.

To determine the molecular pathway required for SLR14-mediated respiratory protection against SARS-CoV-2, we first investigated whether SLR14 affects IFN-I and IFN-III responses in the respiratory tract (Fig. S2 A). Shortly following a single i.v. injection of SLR14, we detected robust levels of IFN-α and IFN-β in the bronchoalveolar lavage fluid (BALF; Fig. 2 A). Consistently, we found substantially elevated expression levels of multiple IFN-I genes, including *Ifna1*, *Ifna2*, *Ifna4*, *Ifna5*, *Ifna7*, *Ifna16*, and *Ifnb1*, in lung tissues of SLR14-treated mice (Fig. 2 B). In contrast, we found no induction of BALF IFN-λ compared with vehicle controls by ELISA and only a mild elevation of *Ifnl2,3* gene expression in the lungs of SLR14-treated mice (Fig. S2, B and C). These results demonstrate that in addition to systemic IFN-I responses as previously reported (Linehan et al., 2018), i.v.-delivered SLR14 rapidly induces local IFN-I production at the respiratory mucosa.

Next, we assessed the effect of IFN-I signaling blockade on SLR14-mediated protection using neutralizing antibodies against the receptor for IFN-I, IFN-α/β receptor (IFNAR). Similar to SLR14 treatment at 4 h after infection, K18-hACE2 mice were completely protected from morbidity and mortality when treated with SLR14 2 h before SARS-CoV-2 infection (Fig. 2, C–E). However, mice that were additionally pretreated with anti-IFNAR antibodies lost the protection provided by SLR14, and all succumbed to the infection by 8 DPI. These results indicated that SLR14-mediated disease protection depends on IFN-I signaling.

Viral infections of the lower respiratory tract are a leading cause of mortality in this disease context, whereas upper respiratory infection primarily contributes to viral transmission. To characterize the tissue sites that are protected by SLR14 and the contribution of IFN-I signaling to SLR14-mediated protection, we collected the lung parenchyma and the trachea to assess viral burden in the lower respiratory tract at 3, 6, and 8 DPI. The ability of SLR14 to suppress lung viral replication was prominent as early as 3 DPI and maintained throughout the course of infection up to 8 DPI. However, the reduction in the level of vRNA in lung tissues was completely abolished when mice were also pretreated with anti-IFNAR antibodies (Fig. 2, F–H). These findings were largely recapitulated in the trachea, although the overall viral titer was lower than that of the lung (Fig. 2, I–K). We also observed a significant decrease in the level of vRNA in nasal washes (Fig. 2 L) and brain tissues (Fig. 2 M) from SLR14-treated mice at 8 DPI. Consistent with reduced vRNA, we found that SLR14-treated mice developed much lower titers of antibodies against SARS-CoV-2 spike protein compared with vehicle- or SLR14 + αIFNAR–treated mice (Fig. 2 N). These results indicated that SLR14 utilizes IFN-I signaling to suppress respiratory and extrapulmonary infection by SARS-CoV-2.

We additionally assessed the role of IFN-I signaling in SLR14-mediated viral control using IFNAR-deficient (*Ifnar*^−/−^) mice. Laboratory mice are not susceptible to SARS-CoV-2 infection due to the inability of the virus to use the mouse orthologue of human ACE2 for viral entry. Therefore, we first transduced C57BL/6J (B6J) or *Ifnar*^−/−^ mice with hACE2-expressing adeno-associated viruses (AAV-hACE2) through intratracheal delivery to sensitize them for SARS-CoV-2 infection (Fig. S2 D; Israelow et al., 2020). Both AAV-hACE2 and K18-hACE2 mice allow for productive replication of SARS-CoV-2 in the lung. K18-hACE2 mice rapidly succumb to intranasal SARS-CoV-2 infection, whereas AAV-hACE2 mice do not manifest apparent disease. 2 wk after transduction, AAV-hACE2 B6J or *Ifnar*^−/−^ mice were infected with SARS-CoV-2 and treated with SLR14 4 h after infection. Consistent with experiments using anti-IFNAR antibodies, AAV-hACE2 *Ifnar*^−/−^ mice did not respond to SLR14 and maintained high levels of vRNA similar to that of untreated controls 4 DPI (Fig. S2 E). In contrast, SLR14-treated AAV-hACE2 B6J mice had significantly reduced level of vRNA compared with untreated controls. Together, these results showed, by two separate approaches, that the SLR14-mediated antiviral resistance against SARS-CoV-2 requires IFNAR.

### SLR14 is taken up by various cell types in the lung

**Figure S3.**
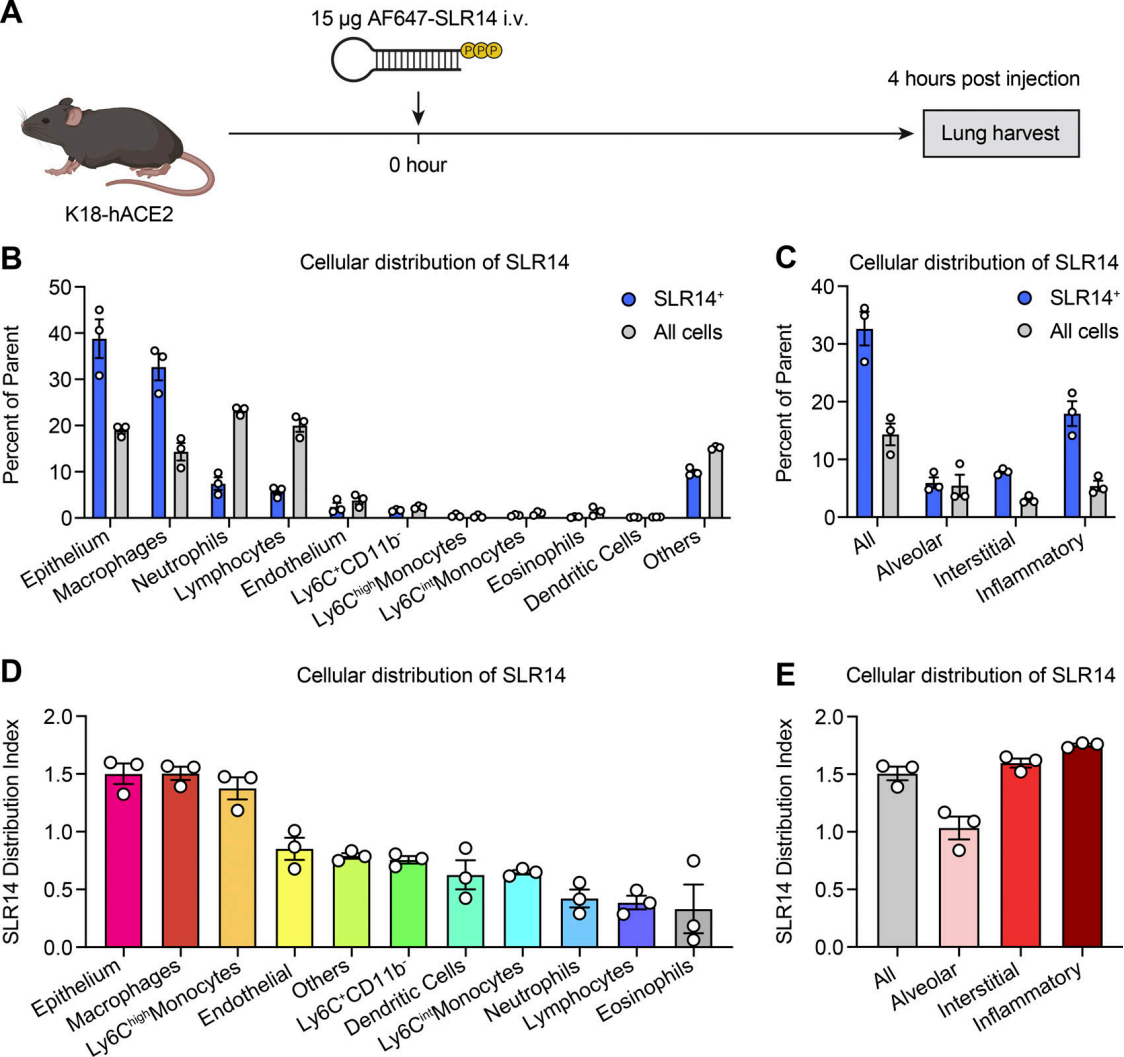
**Intravenously injected SLR14 targets a diverse array of cell types in the lung. (A)** Experimental scheme. K18-hACE2 mice were i.v. injected with 15 µg Alexa Flour 647–conjugated SLR14 or vehicle. Lung tissues were collected for SLR14 uptake analysis by flow cytometry 4 h after injection. Lung tissues from vehicle-injected controls were also collected as negative controls. **(B)** Frequency of indicated immune and nonimmune cell types among SLR14^+^ cells versus total lung cells. **(C)** Frequency of indicated macrophage populations among SLR14^+^ cells or total lung cells. **(D)** Distribution index (frequency of a given cell type in the SLR14^+^ compartment/frequency of all cells) of indicated immune and nonimmune cell types. **(E)** Distribution index of indicated macrophage populations. The specific sets of markers used to identify each subset of cells and assess SLR14 uptake are summarized in Fig. S5. Data are representative of two independent experiments.

RIG-I is ubiquitously expressed in all cell types (Rehwinkel and Gack, 2020). To determine the cell type that is being targeted by polyethyleneimine-complexed SLR14 following i.v. injection and responsible for producing an early source of IFN-I to mediate protection, we injected Alexa Flour 647–conjugated SLR14 into naive K18-hACE2 mice and collected lung tissues 4 h after injection to assess cellular uptake of SLR14 by flow cytometry (Fig. S3 A). Of the total SLR14^+^ cells, we found SLR14 to be broadly distributed across multiple immune and nonimmune cellular compartments (Fig. S3 B). In particular, EpCAM^+^ epithelial cells and CD64^+^ macrophages accounted for the majority of SLR14 uptake (∼70% of SLR14^+^ cells). We further analyzed the composition of SLR14^+^ macrophages and found this population to be mainly CD11b^+^Ly6C^+^ monocyte-derived proinflammatory macrophages, although some SLR14^+^ interstitial and alveolar macrophages were also found (Fig. S3 C). We additionally derived a distribution index to account for cell-type abundance and observed similar patterns of SLR14 uptake by epithelial cells and macrophages (Fig. S3, D and E). Together, these results indicated that i.v.-injected SLR14 is mainly taken up by lung epithelial cells and inflammatory macrophages, contributing to the rapid production of IFN-I and elicitation of local ISG response against SARS-CoV-2 infection.

### SLR14 confers superior protection compared with other IFN-I–based antiviral strategies

**Figure 3.**
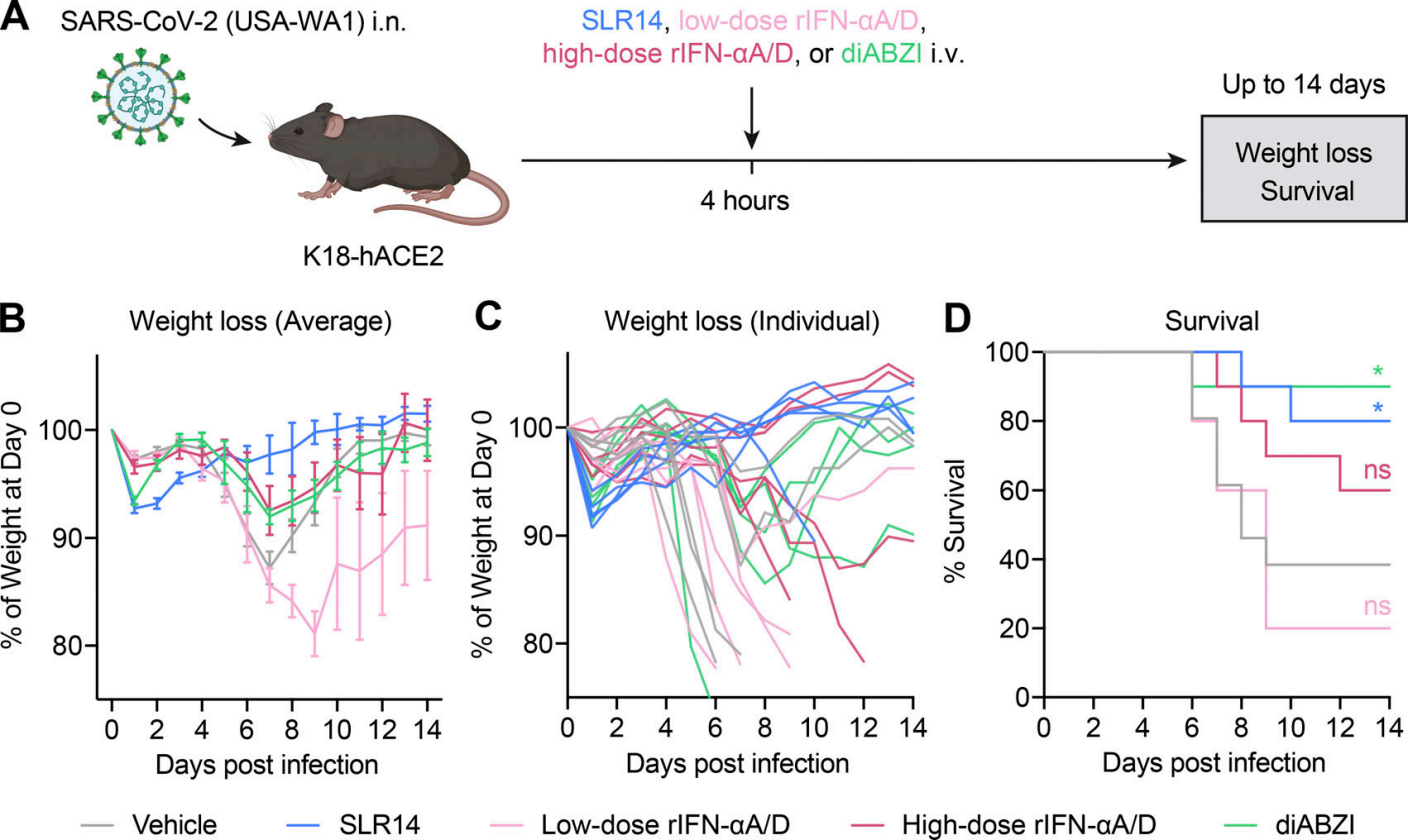
**SLR14 affords superior protection compared to recombinant IFN-I or a STING agonist. (A)** Experimental scheme. K18-hACE2 mice were intranasally infected with 5 × 10^2^ PFU SARS-CoV-2 (2019n-CoV/USA_WA1/2020). 4 h after infection, infected K18-hACE2 mice were i.v. treated with 15 µg SLR14, 2 × 10^4^ U rIFN-αA/D (low-dose), 2 × 10^5^ U rIFN-αA/D (high-dose), 20 µg diABZI, or vehicle. Weight loss and survival were monitored daily up to 14 DPI. Death was recorded when mice were found dead in the cage, moribund, or at 80% of original body weight. **(B–D)** Weight loss and survival of K18-hACE2 mice from 1 to 14 DPI. Mean ± SEM; statistical significance was calculated by log-rank Mantel–Cox test (D); *, P ≤ 0.05. Data are pooled from two independent experiments.

To more thoroughly characterize the antiviral potency of SLR14 against SARS-CoV-2, we also benchmarked our approach against recombinant IFN-I as well as IFN-I-inducing agents in vivo. We focused on a recombinant universal IFN-I (rIFN-αA/D) and a small-molecule agonist, diABZI, that activates STING (a critical component of the cytosolic DNA–sensing pathway) given their promising antiviral activities in preclinical studies (Hoagland et al., 2021; Humphries et al., 2021; Li et al., 2021b). Similar to SLR14 treatment, we treated SARS-CoV-2–infected K18-hACE2 mice i.v. with low-dose rIFN-αA/D, high-dose rIFN-αA/D, or diABZI 4 h after infection and monitored their disease progression (Fig. 3 A). Consistent with our initial observations, SLR14 largely prevented SARS-CoV-2 infection–induced weight loss and lethality (Fig. 3, B–D). rIFN-αA/D treatment resulted in variable but dose-dependent protective effects (Fig. 3, B–D). While high-dose rIFN-αA/D partially alleviated weight loss and lethality in treated K18-hACE2 mice, low-dose rIFN-αA/D failed to confer any protection. The protective capacity of systemic diABZI in preventing lethality was comparable to that of SLR14, although it did not prevent weight loss caused by the infection (Fig. 3, B–D). This was consistent with recent studies reporting diABZI as a highly protective antiviral agent against SARS-CoV-2 infection in mice, especially when given intranasally (Humphries et al., 2021; Li et al., 2021b). Together, these results demonstrate that SLR14 represents a superior antiviral strategy that protects against weight loss and death induced by SARS-CoV-2 infection in vivo.

### SLR14 treatment timing relative to SARS-CoV-2 infection determines protection

**Figure 4.**
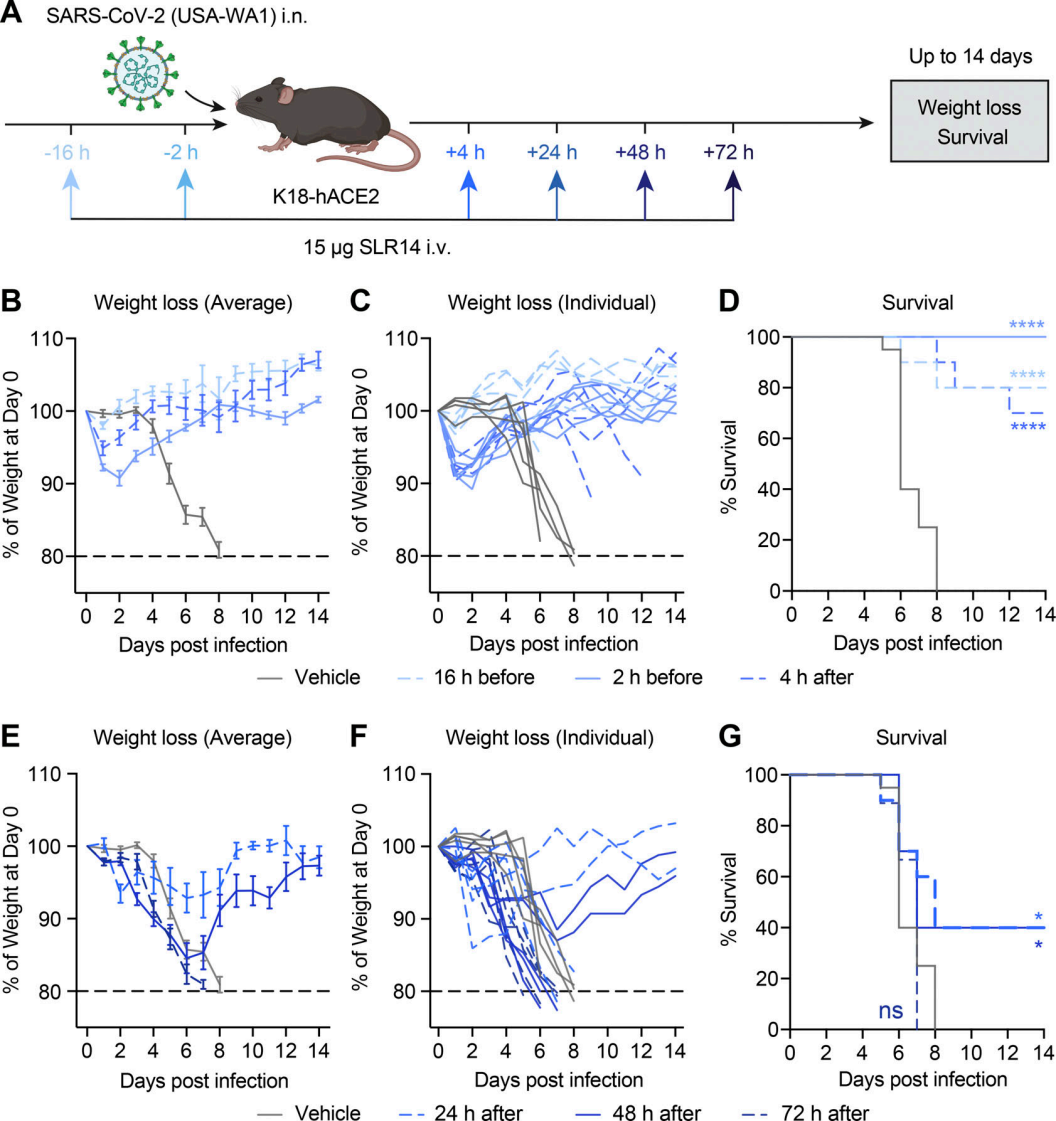
**Protective activities of SLR14 is determined by treatment timing relative to SARS-CoV-2 exposure. (A)** Experimental scheme. K18-hACE2 mice were intranasally infected with 10^3^ PFU SARS-CoV-2 (2019n-CoV/USA_WA1/2020). 15 µg SLR14 was i.v. administered at 16 h before, 2 h before, 4 h after, 24 h after, 48 h after, or 72 h after infection. Weight loss and survival were monitored daily up to 14 DPI. Death was recorded when mice were found dead in the cage, moribund, or at 80% of original body weight. **(B–D)** Weight loss and survival of prophylactically SLR14- and vehicle-treated K18-hACE2 mice from 1 to 14 DPI. **(E–G)** Weight loss and survival of therapeutically SLR14- and vehicle-treated K18-hACE2 mice from 1 to 14 DPI. Mean ± SEM; statistical significance was calculated by log-rank Mantel–Cox test (D and G); *, P ≤ 0.05; ****, P ≤ 0.0001. Data are pooled from of three independent experiments.

Early and robust IFN-I production in response to infection with SARS-CoV-2 is essential for rapid control of viral replication, whereas IFN-I induced late during the infection may contribute to immunopathology and drive severe disease. Thus, we next examined the effect of treatment timing on the protective capacity of SLR14. We treated K18-hACE2 mice with SLR14 at different time points relative to SARS-CoV-2 challenge (Fig. 4 A). Prophylactic treatment of SLR14 either at 16 or 2 h before infection protected mice from weight loss and clinical disease after SARS-CoV-2 infection (Fig. 4, B–D). Similarly, treatment of SLR14 4 h after infection as a post-exposure prophylaxis was also highly protective and largely prevented disease development. However, the efficacy of SLR14 became more dependent on treatment timing when administered therapeutically. Treatment at 24 or 48 h after infection resulted in an intermediate level of protection (40% survival), with some level of morbidity and mortality being observed, while SLR14 lost its protective capacity when administered 72 h after infection (Fig. 4, E–G). These results corroborated the protective role of early IFN-I and, importantly, demonstrated that SLR14-based treatment can be broadly used as prophylaxis and early post-exposure prophylaxis against COVID-19.

### Therapeutic SLR14 cures persistent SARS-CoV-2 infection in immunodeficient mice through induction of IFN-I

**Figure 5.**
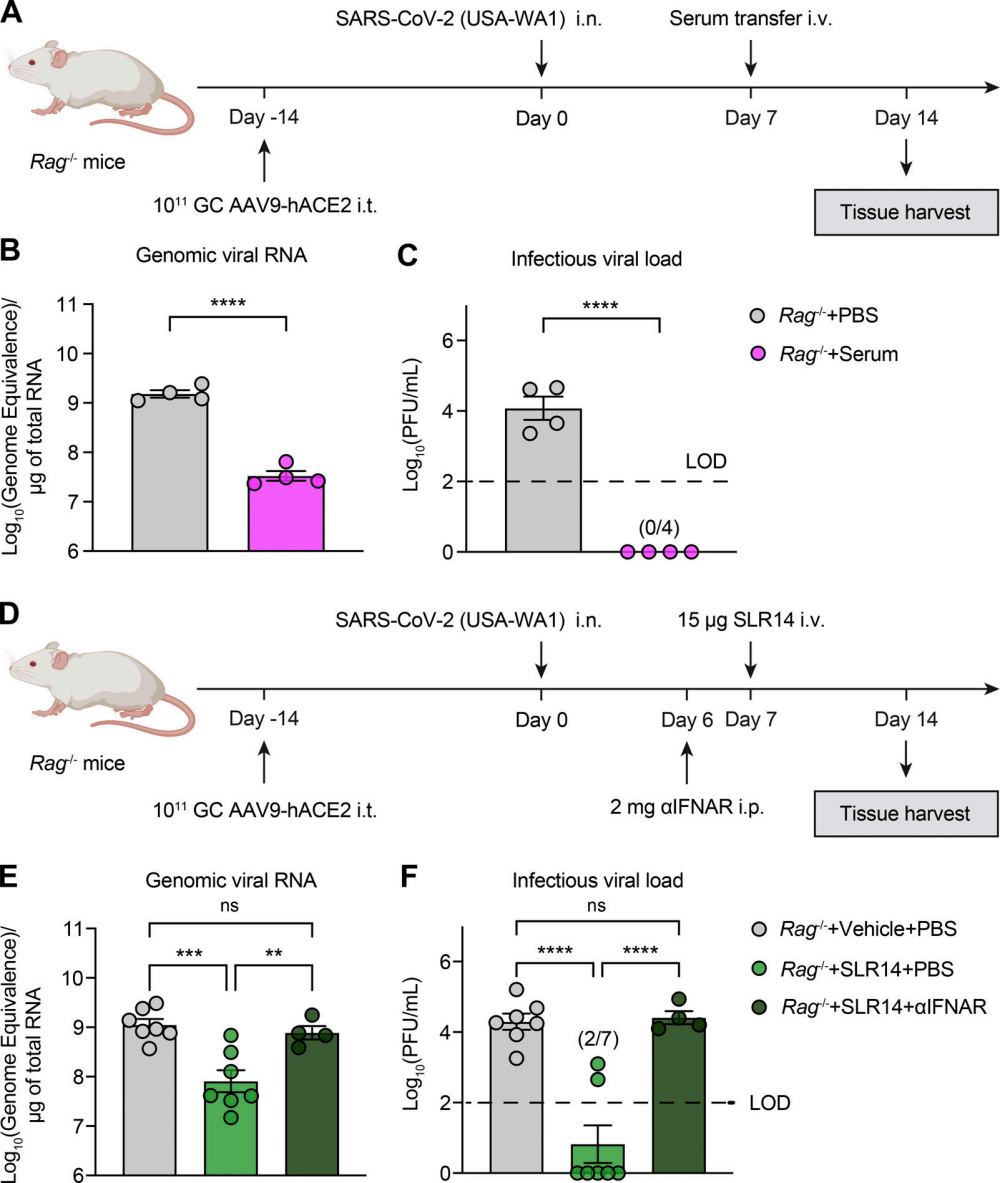
**Therapeutic SLR14 treatment effectively cures persistent SARS-CoV-2 infection in *Rag*^−/−^ mice in an IFN-I–dependent manner. (A)** Experimental scheme. *Rag*^−/−^ mice were intratracheally administered with 10^11^ genome copies of AAV9-hACE2 and allowed to rest for 2 wk before intranasal infection with 10^6^ PFU SARS-CoV-2 (2019n-CoV/USA_WA1/2020). 200 µl convalescent sera or PBS was i.v. administered at 7 DPI. Lung tissues were collected for virological analysis at 14 DPI. **(B)** Measurement of vRNA in the lung at 14 DPI by RT-qPCR. **(C)** Measurement of infectious virus titer in the lung at 14 DPI by plaque assay. **(D)** Experimental scheme. *Rag*^−/−^ mice were intratracheally administered with 10^11^ genome copies of AAV9-hACE2 and allowed to rest for 2 wk before intranasal infection with 10^6^ PFU SARS-CoV-2 (2019n-CoV/USA_WA1/2020). 15 µg SLR14 or vehicle was i.v. administered at 7 DPI. 24 h before SLR14 injection, half of the SLR14-treated mice were additionally given 2 mg anti-IFNAR antibodies. Lung tissues were collected for virological analysis at 14 DPI. **(E)** Measurement of vRNA in the lung at 14 DPI by RT-qPCR. **(F)** Measurement of infectious virus in the lung 14 at DPI by plaque assay. Limit of detection (LOD), 10^2^ PFU/ml. Mean ± SEM; statistical significance was calculated by one-way ANOVA followed by Tukey correction (B, C, E, and F); **, P ≤ 0.01; ***, P ≤ 0.001; ****, P ≤ 0.0001. Data are pooled from two independent experiments.

There is a clinically unmet need for the development of an effective therapy to treat chronic SARS-CoV-2 infection in immunodeficient individuals and prevent further emergence of viral variants. We have previously demonstrated that AAV-hACE2–transduced *Rag1*^−/−^ or *Rag2*^−/−^ mice (which completely lack mature T and B cells, collectively referred to as *Rag*^−/−^ mice) become chronically infected following SARS-CoV-2 infection, similar to what is seen in immunodeficient patients (Israelow et al., 2021). These mice maintain stable levels of viral RNA and infectious virus for at least 14 DPI. This is in stark contrast to B6J mice, which clear the infection by 7 DPI and remain virus-free thereafter. Given that CP therapy has been implemented to treat immunocompromised patients with COVID-19 (Hueso et al., 2020), we first validated whether persistently infected *Rag*^−/−^ mice are a clinically relevant model in their response to CP therapy. To this end, we adoptively transferred sera from convalescent AAV-hACE2 B6J mice into persistently infected recipient AAV-hACE2 *Rag*^−/−^ mice 7 DPI and measured lung viral titer 14 DPI (Fig. 5 A). We found that CP transfer resulted in significant reduction in vRNA and complete clearance of infectious virus in the lung compared with PBS-treated SARS-CoV-2 infected AAV-hACE2 *Rag*^−/−^ controls (Fig. 5, B and C). These results suggest that *Rag*^−/−^ mice are a suitable in vivo model of immunocompromised patients for preclinical testing of antiviral therapeutics, as they support persistent SARS-CoV-2 infection and derive benefits from CP therapy.

We next examined whether SLR14 can be used as a therapeutic modality to treat persistent infection in *Rag*^−/−^ mice. We infected AAV-hACE2 *Rag*^−/−^ mice with SARS-CoV-2, treated them with SLR14 7 DPI, and collected lung tissues 14 DPI to assess the viral burden (Fig. 5 D). We injected SLR14 at 7 DPI instead of administering immediately following viral exposure so that chronic infection could be established in AAV-hACE2 *Rag*^−/−^ mice first, before any intervention was provided. Additionally, unlike K18-hACE2 mice, which we have shown are only effectively protected from preexposure or early postexposure intervention due to their rapid disease progression (Fig. 4), AAV-hACE2 mice do not die from intranasal SARS-CoV-2 infection, allowing treatments to be given at much later time points. 1 d before SLR14 treatment, a subset of SLR14-treated AAV-hACE2 mice also received anti-IFNAR antibodies. SLR14 treatment led to a significant reduction in the level of lung vRNA (Fig. 5 E). The ability of SLR14 in decreasing vRNA was abolished when anti-IFNAR blocking antibody was given, suggesting SLR14 similarly utilizes IFN-I signaling to promote viral clearance in mice lacking the adaptive immune system. We additionally observed a striking difference in the infectious viral load in lung tissues from SLR14-treated mice compared with vehicle controls. Treatment with SLR14, but not vehicle, significantly reduced viral burden and resulted in complete clearance of infectious virus in five out of seven AAV-hACE2 *Rag*^−/−^ mice and reduction of viral titer in the remaining two (Fig. 5 F). Moreover, SLR14-mediated protection required IFNAR signaling. These results show that in the setting of complete T and B cell deficiency, a single therapeutic SLR14 treatment, through the induction of IFN-I, is sufficient to cure persistent infection.

### SLR14 affords broad protection against immunologically evasive SARS-CoV-2 variants

As SARS-CoV-2 variants continue to emerge and spread, antiviral therapeutics that confer broadly cross-reactive protection are urgently needed. Emerging evidence suggests that several variants have acquired mutations that confer elevated resistance to IFN-I treatment in cell culture (Guo et al., 2021 *Preprint*; Thorne et al., 2021 *Preprint*). However, whether such altered properties in vitro translate into evasion of IFN-based therapy in vivo remains unclear. To this end, we obtained five clinically relevant SARS-CoV-2 variants of concern (VOCs) or variants of interest, including B.1.1.7, B.1.351, P.1, B.1.526, and B.1.617.2, and used them to infect K18-hACE2 mice. The P.1, B.1.526, B.1.1.7, and B.1.617.2 variants were identified and isolated as a part of the Yale SARS-CoV-2 Genomic Surveillance Initiatives (Kalinich et al., 2020), and the B.1.351 variant was obtained from BEI Resources Repository. All variants were confirmed to harbor signature mutations characteristic of their respective lineages and show correct placement in the phylogenetic tree built with public SARS-CoV-2 genomic sequences (Table S1; Lucas et al., 2021).

**Figure 6.**
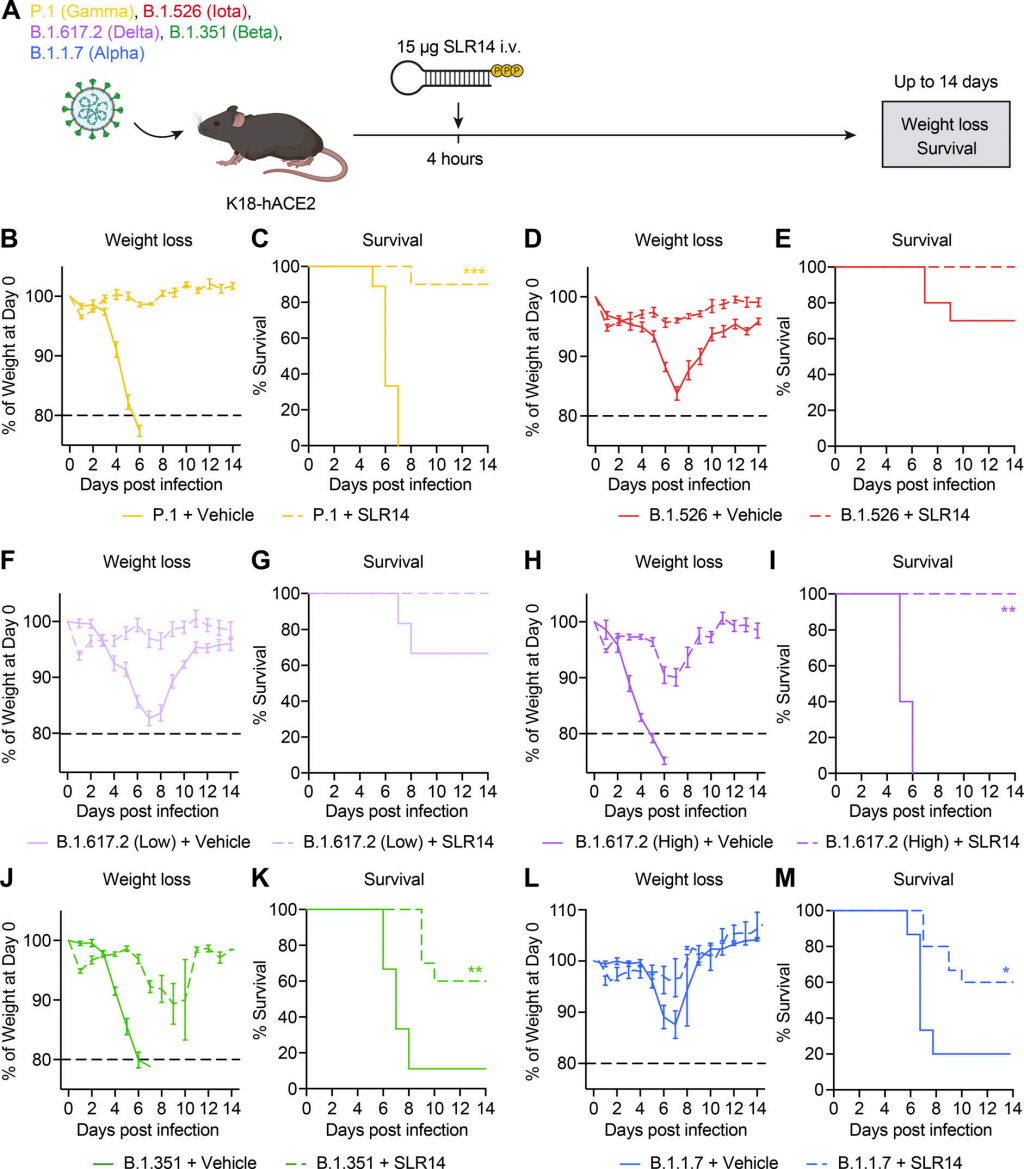
**SLR14 is broadly protective against emerging immunologically evasive SARS-CoV-2 variants. (A)** Experimental scheme. K18-hACE2 mice were intranasally infected with P.1 (Gamma), B.1.526 (Iota), B.1.617.2 (Delta), B.1.351 (Beta), or B.1.1.7 (Alpha) variant. 15 µg SLR14 or vehicle was i.v. administered at 4 h after infection. Weight loss and survival were monitored daily up to 14 DPI. Death was recorded when mice were found dead in the cage, moribund, or at 80% of original body weight. **(B and C)** Weight loss and survival of SLR14- and vehicle-treated K18-hACE2 mice from 1 to 14 DPI following 10^4^ PFU P.1 infection. **(D and E)** Weight loss and survival of SLR14- and vehicle-treated K18-hACE2 mice from 1 to 14 DPI following 10^4^ PFU B.1.526 infection. **(F and G)** Weight loss and survival of SLR14- and vehicle-treated K18-hACE2 mice from 1 to 14 DPI following 5 × 10^5^ PFU (low-dose) B.1.617.2 infection. **(H and I)** Weight loss and survival of SLR14- and vehicle-treated K18-hACE2 mice from 1 to 14 DPI following 5 × 10^7^ PFU (high-dose) B.1.617.2 infection. **(J and K)** Weight loss and survival of SLR14- and vehicle-treated K18-hACE2 mice from 1 to 14 DPI following 10^4^ PFU B.1.351 infection. **(L and M)** Weight loss and survival of SLR14- and vehicle-treated K18-hACE2 mice from 1 to 14 DPI following 10^4^ PFU B.1.1.7 infection. Mean ± SEM; statistical significance was calculated by log-rank Mantel–Cox test (C, E, G, I, K, and M); *, P ≤ 0.05; **, P ≤ 0.01; ***, P ≤ 0.001. Data are pooled from two independent experiments.

**Figure S4.**
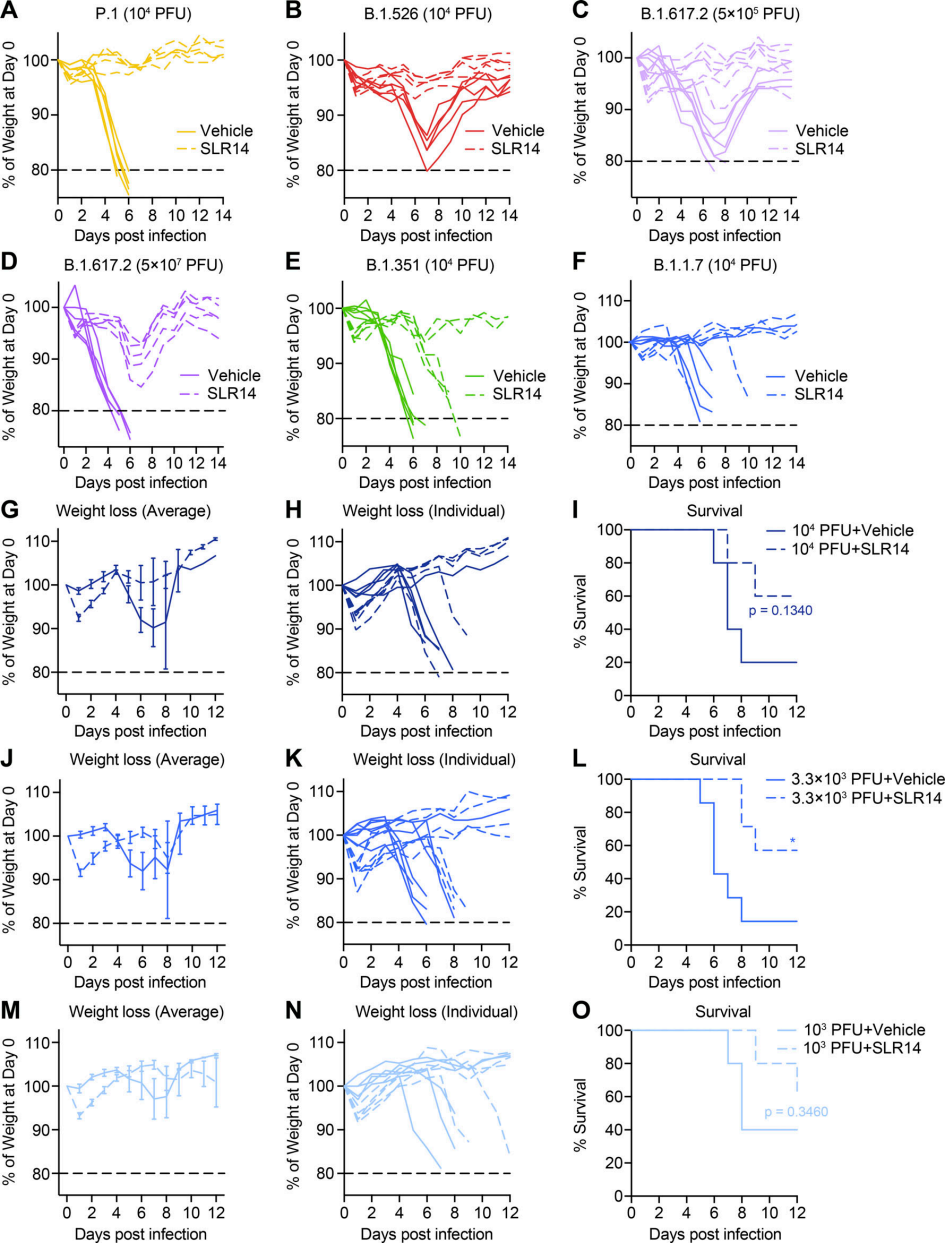
**Alpha variant exhibits partial therapeutic resistance to SLR14 treatment. (A–F)** Experimental scheme. K18-hACE2 mice were intranasally infected with P.1 (Gamma), B.1.526 (Iota), B.1.617.2 (Delta), B.1.351 (Beta), or B.1.1.7 (Alpha) variant. 15 µg SLR14 or vehicle was i.v. administered at 4 h after infection. Weight loss by individual SLR14- and vehicle-treated K18-hACE2 mice from 1 to 14 DPI following infection with P.1 (A), B.1.526 (B), low-dose B.1.617.2 (C), high-dose B.1.617.2 (D), B.1.351 (E), or B.1.1.7 (F). Data are representative of two independent experiments. **(G–O)** Experimental scheme. K18-hACE2 mice were intranasally infected with 10^4^ PFU, 3.3 × 10^3^ PFU, or 10^3^ PFU B.1.1.7 variant. 15 µg SLR14 or vehicle were i.v. administered at 4 h after infection. Weight loss and survival were monitored daily up to 14 DPI. Death was recorded when mice were found dead in the cage, moribund, or at 80% of original body weight. **(G–I)** Weight loss and survival of SLR14- and vehicle-treated K18-hACE2 mice from 1 to 14 DPI following infection with 10^4^ PFU B.1.1.7. **(J–L)** Weight loss and survival of SLR14- and vehicle-treated K18-hACE2 mice from 1 to 14 DPI following infection with 3.3 × 10^3^ PFU B.1.1.7. **(M–O)** Weight loss and survival of SLR14- and vehicle-treated K18-hACE2 mice from 1 to 14 DPI following infection with 10^3^ PFU B.1.1.7. Mean ± SEM; statistical significance was calculated by log-rank Mantel–Cox test (I, L, and O); *, P ≤ 0.05. Data are representative of or pooled from two independent experiments.

To examine whether SLR14 is protective against SARS-CoV-2 variants, we infected mice with variants P.1, B.1.526, B.1.617.2, B.1.351, or B.1.1.7 and treated them with SLR14 4 h after infection (Fig. 6 A). SLR14 afforded potent protection against the P.1 variant, almost completely preventing morbidity and mortality in the face of highly lethal infection, even when treated post-exposure prophylactically (Fig. 6, B and C; and Fig. S4 A). SLR14 also fully prevented weight loss or any discernable disease following infection with B.1.526, which, in untreated mice, caused a less pathogenic infection compared with that of the ancestral strain or circulating variants (Fig. 6, D and E; and Fig. S4 B). In addition, we also found that SLR14 was highly effective against B.1.617.2, protecting against weight loss in K18-hACE2 mice infected with a relatively low dose of virus (Fig. 6, F and G; and Fig. S4 C). In contrast, vehicle-treated mice uniformly lost 10–20% of their starting body weight. Remarkably, even in the face of a high-dose infection, SLR14-treated mice were protected from clinical disease or death, whereas vehicle controls rapidly succumbed to the infection (Fig. 6, H and I; and Fig. S4 D). Consistent with the reported resistance to IFN-I signaling in vitro (Guo et al., 2021 *Preprint*; Thorne et al., 2021 *Preprint*), SLR14 treatment was less effective against infection with B.1.351 or B.1.1.7 in vivo, conferring ∼40–50% net protection in K18-hACE2 mice (60% survival in SLR14-treated mice compared with 10–20% survival in vehicle controls; Fig. 6, J–M; Fig. S4, E and F). Additional experiments with the B.1.1.7 variant confirmed its partial resistance to SLR14 treatment, irrespective of initial sizes of viral inoculum (Fig. S4, G–O). Nevertheless, clear benefits were seen with prophylactic treatment of SLR14. These results suggest that SLR14 confers broad-coverage protection against antibody- and IFN-I–evasive variants.

## Discussion

The sudden arrival and devastating spread of COVID-19 have emphasized the importance of continuous efforts to develop broad-spectrum antiviral agents. Here, we examined the in vivo efficacy of SLR14 against viral replication throughout the respiratory tract and disease development in a mouse model of severe SARS-CoV-2 infection. We first showed that SLR14 rapidly triggers local production of IFN-I in the respiratory tract. Consistent with the induction of airway IFN-I responses, we found that SLR14 conferred considerable antiviral resistance in the lower respiratory tract and effectively prevented morbidity and mortality following infection with the ancestral virus. We also examined the effect of host factor, tissue compartment, and treatment timing in the protective capacity of SLR14, and we found that the protective efficacy of SLR14 depends on intact IFNAR signaling and that early SLR14 administration provided superior protection, while treatment as late as 48 h after infection still afforded partial protection. We further tested the therapeutic potential for SLR14 in chronically infected immunodeficient mice and demonstrated that a single dose of SLR14 conferred near-sterilizing immunity by the innate immune system alone, even in the absence of T and B cells. Finally, we found that SLR14 confers broad protection against all emerging SARS-CoV-2 variants.

The apparent protective role of early and regulated IFN-I suggests IFN-based therapies can be used for prevention and treatment of COVID-19. In a golden hamster model of SARS-CoV-2 infection, intranasal administration of commercially available universal IFN (rIFN-αA/D) reduced viral burden and attenuated pathology in the lung (Hoagland et al., 2021). In a retrospective multicenter cohort study of 446 COVID-19 patients, early administration of inhaled IFNα2b produced more favorable clinical responses compared with lopinavir/ritonavir treatment alone and was associated with reduced in-hospital mortality (Wang et al., 2020). While results from rIFN-based clinical trials are promising, one of the major disadvantages of this approach is its high cost, with direct medical costs ranging between $1,120 and $1,962 for the IFN treatment regimen and $2,156 and $5,887 for the PEG-IFN treatment regimen (Nguyen et al., 2020). In addition, administration of rIFN has been shown to induce neutralizing antibody response against IFN that could render the therapy ineffective (Giovannoni et al., 2002; Matsuda et al., 2012). SLR14 addresses these challenges with 1) increased affordability due to its synthetic simplicity, small size, and manufacturability, and 2) its ability to induce different members of the IFN-I family, including 10 IFN-α subtypes and an IFN-β, which maximizes the likelihood of downstream responses to be functional (Linehan et al., 2018). In particular, the ability of SLR14 to elicit IFN-β is ideal, as it enables early medical intervention for COVID-19 patients with preexisting autoantibodies against one or multiple subtypes of IFN-α, who are particularly susceptible to prolonged viral replication and severe disease after infection with SARS-CoV-2 (Meffre and Iwasaki, 2020). Besides rIFN, innate modulators that induce IFN production, such as poly(I:C) and diABZI, may also serve as a strategy to combat COVID-19. We have previously found that both SLRs and poly(I:C) induce a diverse array of genes associated with antiviral immunity. Importantly, SLRs induced an IFN-I–dominant response, both systemically (Linehan et al., 2018) and in mucosal tissues (this study). When administered i.v. in mice, SLR14 induced a much stronger systemic IFN-α response compared with that of poly(I:C) (Linehan et al., 2018). Given that SLR14-mediated protection critically depends on IFN-I, these data indicate that SLR14 will be similarly, if not more, effective against SARS-CoV-2 than poly(I:C). With their potent antiviral activities, however, the safety profile related to inflammation associated with systemic administration of rIFN-I– and IFN-I–inducing agents (including SLR14), particularly in the context of SARS-CoV-2 infection, needs to be carefully examined in future studies.

The clinical efficacy of CP in patients with severe COVID-19 has not been thoroughly demonstrated, and its use in different stages of infection and disease remains experimental (Li et al., 2020; Simonovich et al., 2021). Emergence of immune-evading variants from patients with immunosuppression of T cell and B cell arms indicate caution should be used for CP therapy (Kemp et al., 2021). In these patients, the administered antibodies have little support from cytotoxic CD8 T cells or helper CD4 T cells, thereby reducing the chances of clearance and theoretically allowing for SARS-CoV-2 escape. Therefore, a novel therapeutic paradigm that treats persistent viral infection regardless of its effect on adaptive immunity will hold immense potential for this patient population. We have shown in this study that a single dose of SLR14 in mice lacking the adaptive immune system in the setting of chronic SARS-CoV-2 infection can induce near-sterilizing immunity. These results demonstrated that SLR14’s utility extends beyond prophylactic antivirals, but also therapeutics that can be given to patients with immunocompromised conditions, providing an immediate solution to simultaneously cure chronic infection and suppress future emergence of immune-evasive variants. From an evolutionary perspective, such sterilizing protection induced exclusively through innate immune activation is analogous to antiviral mechanisms in metazoan organisms lacking adaptive immunity, which provides a basic, yet crucial, protective strategy against viral pathogens (Wang et al., 2015).

Vaccines remain the best approach to thwart the COVID-19 pandemic. However, with many countries lacking access to adequate vaccine doses, alternative strategies need to be developed and rapidly distributed to parts of the world severely impacted by these variants. Here, we showed that SLR14 potently prevented morbidity and mortality following infection with clinically relevant VOC, which have vastly different signature mutations and immune-evading capacity. Consistent with a recent study examining IFN-I potency against different variants in vitro (Guo et al., 2021 *Preprint*; Thorne et al., 2021 *Preprint*), the protective capacity of SLR14 was less impressive when administered against B.1.351 or B.1.1.7. Importantly, SLR14 still retained considerable residual antiviral capacity, which may be attributed by the speed, magnitude, and diversity of IFN-I responses induced by SLR14 that could collectively overcome viral resistance (Linehan et al., 2018). Up until this point, B.1.1.7 has been recognized as a minimally immune-evasive variant based on both antibodies and T cell recognition. Here, we provide the first set of in vivo evidence to suggest that B.1.1.7 exhibits signs of IFN-I evasion and responds only moderately to IFN-based therapy. Such innate immune evasion may underlie the rapid global spread of B.1.1.7. These results showcase SLR14’s ability to be used not only as a therapeutic agent but also as an investigative tool for functional assessment of basic SARS-CoV-2 biology.

Several drugs have been approved by the US Food and Drug Administration under Emergency Use Authorization, including dexamethasone, remdesivir, monoclonal antibodies, tocilizumab, and baricitinib, to treat COVID-19 (Beigel et al., 2020; Calabrese and Calabrese, 2021; Horby et al., 2021; Kalil et al., 2021; Rosas et al., 2021; Taylor et al., 2021). However, these therapeutics typically provide modest benefits at best and are limited to a subset of patients. While currently licensed vaccines demonstrate astounding protective efficacy against COVID-19, a new variant may develop in the future to significantly reduce efficacy. Further, there is a global shortage in vaccines with inequitable access in many lower income countries. The development, characterization, and ultimate deployment of an effective antiviral against SARS-CoV-2 could prevent substantial morbidity and mortality associated with COVID-19. In addition to its therapeutic potential, SLR14 can be used as an invaluable investigative tool to advance our understanding of protective antiviral immunity against respiratory viruses, which will enable the rational design of next-generation antiviral therapeutics. Lastly, with the prevalence of prepandemic zoonotic viruses and the unpredictable overlap of human and wild animal ecologies, the potential for a novel viral emergence from its natural reservoir into humans is a matter of time. Through the creation of a simple and versatile RNA-based therapeutics, our studies will facilitate pandemic preparedness and response against future respiratory pathogens.

## Materials and methods

### Ethics

The institutional review board of the Yale University Human Research Protection Program determined that the RT-qPCR testing and sequencing of deidentified remnant COVID-19 clinical samples conducted in this study is not research involving human subjects (institutional review board protocol ID 2000028599).

### Mice

B6.Cg-Tg(K18-ACE2)2Prlmn/J (K18-hACE2), B6(Cg)-Ifnar1^tm1.2Ees^/J (Ifnar1^−/−^), B6.129S7-*Rag1^tm1Mom^*/J (B6J *Rag1*^−/−^), and C.129S7(B6)-*Rag1^tm1Mom^*/J (BALB/c *Rag1*^−/−^) mice were purchased from The Jackson Laboratory and subsequently bred and housed at Yale University. *Rag2*^−/−^ mice were generously gifted from R. Flavell (Yale University, New Haven, CT). 6- to 10-wk-old mixed-sex mice were used throughout the study. All mice were housed as groups of five or six individuals per cage and maintained on a 12-h light/dark cycle (lights on at 7:00 a.m.) at 22–25°C temperature and 30–70% relative humidity under specific pathogen–free conditions. All mice were fed with regular rodent’s chow and sterilized water ad libitum. All procedures used in this study (sex-matched and age-matched) complied with federal guidelines and the institutional policies of the Yale School of Medicine Animal Care and Use Committee.

### Virus sequencing

Nucleic acid was extracted from 300 µl viral transport medium from nasopharyngeal swabs and eluted in 75 µl using the MagMAX viral/pathogen nucleic acid isolation kit. Extracted nucleic acid was tested by our multiplexed RT-qPCR variant assay (Vogels et al., 2021a; Vogels et al., 2021b), and then libraries were prepared using the Illumina COVIDSeq Test RUO version. The protocol was slightly modified by lowering the annealing temperature of the amplicon PCR step to 63°C and reducing tagmentation to 3 min. Pooled libraries were sequenced on the Illumina NovaSeq (paired-end 150). Data were processed and consensus sequences were generated using iVar (version 1.3.1) with the minimum depth threshold (-m) at 20 and minimum frequency threshold (-t) at 0.6 (Grubaugh et al., 2019). Genome sequences were uploaded to GISAID. Samples belonging to the B.1.1.7 (EPI_ISL_1038987), P.1 (EPI_ISL_1293215), B.1.526 (EPI_ISL_944591), and B.1.617.2 (EPI_ISL_2035068) lineages were selected for virus isolation from the original sample. Virus belonging to the B.1.351 lineage was obtained from BEI Resources.

### Virus isolation

Samples selected for virus isolation were diluted 1:10 in DMEM and then filtered through a 45-µM filter. The samples were tenfold serially diluted from 1:50 to 1:19,531,250. The dilution was subsequently incubated with TMPRSS2-Vero E6 in a 96-well plate and adsorbed for 1 h at 37°C. After adsorption, replacement medium was added, and cells were incubated at 37°C for up to 5 d. Supernatants from cell cultures with cytopathic effect were collected, frozen, thawed, and subjected to RT-qPCR. Fresh cultures were inoculated with the lysates as described above for viral expansion. Viral infection was subsequently confirmed through reduction of cycle threshold values in the cell cultures with the multiplex variant qPCR assay. Expanded viruses were resequenced following the same method as described above and were identical to the original clinical sample sequence. Genome sequences of cultured viruses B.1.1.7 (SARS-CoV-2/human/USA/Yale-3363/2021; GenBank accession: MZ202178), B.1.351 (SARS-CoV-2/human/ZAF/Yale-3366/2020; GenBank accession: MZ202314), P.1 (SARS-CoV-2/human/USA/Yale-3365/2021; GenBank accession: MZ202306), B.1.526 (SARS-CoV-2/human/USA/Yale-3362/2021; GenBank accession: MZ201303), and B.1.617.2 (SARS-CoV-2/human/USA/Yale-5641/2021; GenBank accession: MZ468047) were uploaded to GenBank. We used Nextclade v0.14.2 (https://clades.nextstrain.org/) to generate a phylogenetic tree and to compile a list of amino acid changes in the virus isolates as compared with the Wuhan-Hu-1 reference strain (Table S1).

### Synthesis, purification, and labeling of the SLR14 oligonucleotide

The triphosphorylated RNA oligonucleotides SLR14 (5′-pppGGAUCGAUCGAUCGUUCGCGAUCGAUCGAUCC-3′) and SLR14-amino (5′-pppGGAUCGAUCGAUCGUXCGCGAUCGAUCGAUCC-3′, where X = aminomodifier C6dT; Glen Research) were prepared as described previously (Jiang et al., 2019). Briefly, for every 1 mg of starting material, removal of the oligonucleotide from the polymer support and base deprotection was performed in a 1:1 mixture of 40% methylamine (Sigma-Aldrich) and 30% ammonium hydroxide (JT Baker) at 65°C for 15 min. The solution was cooled on ice for 10 min, transferred to a new vial, and evaporated to dryness. 500 µl of absolute ethanol was added, and the mixture was evaporated to dryness again. To deprotect the 2′-OH groups, the dry oligonucleotide was incubated with 500 µl of a 1 M solution of tetrabutylammonium fluoride in tetrahydrofuran (Sigma-Aldrich) at room temperature for 36 h. 500 µl of 2 M sodium acetate (pH 6.0) was added, and the solution was evaporated to a 500–600 µl volume, extracted with 3 × 800 µl ethyl acetate, and ethanol precipitated. The RNA oligonucleotide was then purified on a 16% denaturing polyacrylamide gel. For fluorescent labeling, for every 1 mg of starting material, the purified SLR14-amino oligonucleotide was dissolved in 200 µl of 0.25 M sodium bicarbonate buffer (pH 9.2). Then, a solution containing 0.5 mg Alexa Fluor 647 NHS ester (Life Technologies) in 200 µl N,N-dimethylformamide was added, and the reaction mixture was incubated at room temperature for 2 h. The labeled oligonucleotide (AF647-SLR14) was ethanol precipitated and purified on a 20% denaturing polyacrylamide gel.

### In vivo SARS-CoV-2 infection

Before infection, mice were anesthetized using 30% (vol/vol) isoflurane diluted in propylene glycol. For K18-hACE2 mice, 50 µl of SARS-CoV-2 was delivered intranasally at 10^3^ PFU per mouse, unless specified otherwise. Following infection, weight loss and survival were monitored daily up to 14 DPI. For AAV-hACE2 mice, 50 µl SARS-CoV-2 was delivered intranasally at 10^6^ PFU per mouse. Experiments involving SARS-CoV-2 infection were performed in a biosafety level 3 facility with approval from the Yale Institutional Animal Care and Use Committee and Yale Environmental Health and Safety.

### Antibody and drug treatment in mice

For IFNAR blockade, mice were treated once with 2 mg blocking antibodies diluted in 200 µl PBS 1 d before infection (Clone MAR1-5A3; BioXCell). Universal IFN-I (rIFN-αA/D, no. 11200; PBL Assay Science) was supplied frozen in PBS containing 0.1% BSA. Cross-species activity of rIFN-αA/D on mouse cells was confirmed by the manufacturer and a previous study (Uccellini and García-Sastre, 2018). 4 h after infection, 2 × 10^4^ U rIFN-αA/D (low-dose; 10^6^ U/kg) or 2 × 10^5^ U rIFN-αA/D (high-dose; 10^7^ U/kg) were diluted in 100 µl PBS and i.v. administered to mice. STING agonist diABZI (Compound 3; Selleckchem) was first reconstituted in DMSO at 50 mg/ml. 20 µg diABZI (1 mg/kg) was diluted in 100 µl PBS and i.v. administered to mice 4 h after infection. The dosage for systemic diABZI treatment was determined based on previous publications (Humphries et al., 2021; Ramanjulu et al., 2018). The dosing solution was prepared fresh and confirmed to be clear at the time of administration.

### i.v. injection of SLR14 in mice

At indicated time points, 15 µg SLR14 was i.v. injected. Briefly, 15 µg (∼0.75 mg/kg body weight) SLR14 and 4 µl jetPEI (Polyplus Transfection) were diluted and mixed with 5% glucose solution to a total of 100 µl injection solution per mouse. After 15 min of incubation at room temperature, the 100-µl complex was carefully injected into the retro-orbital sinus with a 0.5-ml BD insulin syringe. Before injection, mice were anesthetized using 30% (vol/vol) isoflurane diluted in propylene glycol. H_2_O and jetPEI were mixed with 5% glucose solution and used as a vehicle control.

### AAV-hACE2 transduction

AAV-hACE2 transduction was performed as previously described (Israelow et al., 2020). AAV9 vector encoding hACE2 was purchased from Vector Biolabs (AAV9-CMV-hACE2). Animals were anaesthetized using a mixture of ketamine (50 mg/kg) and xylazine (5 mg/kg) injected intraperitoneally. The rostral neck was shaved and disinfected. A 5-mm incision was made, the salivary glands were retracted, and the trachea was visualized. Using a 32-G insulin syringe, a 50-µl bolus injection of 10^11^ genomic copies AAV-CMV-hACE2 was injected into the trachea. The incision was closed with 4–0 Vicryl suture. Following intramuscular administration of analgesic (meloxicam and buprenorphine, 1 mg/kg), animals were placed on a heating pad and closely monitored until full recovery.

### Measurements of genomic RNA and infectious virus

Viral RNA and titer from mouse lung tissues were measured as previously described (Israelow et al., 2020). Briefly, at indicated time points, mice were euthanized with 100% isoflurane. The whole lung was placed in a Lysing Matrix D tube (MP Biomedicals) with 1 ml PBS and homogenized using a table-top homogenizer at medium speed for 2 min. For RNA analysis, 250 µl of the lung homogenates was added to 750 µl Trizol LS (Invitrogen), and RNA was extracted with the RNeasy Mini Kit (Qiagen) according to the manufacturer’s instructions. SARS-CoV-2 RNA levels were quantified with 250 ng RNA inputs using the Luna Universal Probe One-Step RT-qPCR Kit (New England Biolabs) using real-time RT-PCR primer/probe sets 2019-nCoV_N1 (CDCN1) and 2019-nCoV_N2 (CDCN2). For determination of infectious titer, plaque assays were performed using lung homogenates in Vero E6 cells cultured with MEM supplemented with NaHCO_3_, 4% FBS, and 0.6% Avicel RC-581. 48 h after infection, plaques were resolved by 1-h fixation with 10% formaldehyde and sequential 1-h staining with 0.5% crystal violet in 20% ethanol. Finally, plates were rinsed in water for better visualization of plaques.

### Measurements of cellular gene expression by RT-qPCR

Following RNA extraction, total cDNA was prepared with 2 µg RNA inputs using the iScript cDNA Synthesis Kit following the manufacturer’s instruction (Bio-Rad). RT-PCR was then performed using the SYBR Green PCR Master Mix (Applied Biosystems). For each RT-qPCR reaction, 50 ng cDNA input was used.

### Immunohistochemistry

Mice were first perfused by intracardiac injection 20 ml PBS into the right ventricle until the lung appeared opaque. After perfusion, the lung was slowly inflated with 1 ml 4% paraformaldehyde (PFA; Electron Microscopy Sciences) through intratracheal instillation. Following inflation, the trachea was quickly tied with suture. Tissue was collected and fixed in 4% PFA overnight. Yale Pathology kindly assisted with embedding, sectioning, and H&E staining of lung tissues. H&E-stained lung sections were then imaged by a fluorescence microscope (BX51; Olympus) with a 10× lens.

### BALF collection

Mice were euthanized with 100% isoflurane. After euthanasia, the trachea was exposed and the lung was slowly inflated with 1 ml PBS through intratracheal instillation. Lung tissues were flushed three times. Following lavage, samples were centrifuged at 3,900 rpm for 5 min at 4°C; the supernatant (i.e., BALF) was aliquoted in 100 µl aliquots and stored at −80°C.

### Antibodies for flow cytometry

Anti-mouse antibodies used in this study, together with vendors and dilutions, are listed as follows: FITC anti-mCD11c (N418; 1:400; BioLegend), PerCP-Cy5.5 anti-mLy6C (HK1.4; 1:400; BioLegend), Alexa Fluor 700 anti-mLy6G (1A8; 1:400; BioLegend), Brilliant Violet 786 anti-mCD11b (M1/70; 1:400; BioLegend), APC-Cy7 anti-mTCRb (H57-597; 1:400; BioLegend), APC-Cy7 anti-mCD3 (H57-597; 1:400; BioLegend), APC-Cy7 anti-mCD19 (6D5; 1:400; BioLegend), APC-Cy7 anti-mNK1.1 (PK136; 1:400; BioLegend), PE anti-mCD64 (X54-5/7.1; 1:200; BioLegend), Brilliant Violet 711 anti-mSiglecF (E50-2440; 1:200; BD Biosciences), and Pacific Blue anti-mI-A/I-E (M5/114.15.2; 1:400; BioLegend).

### Flow cytometry

**Figure S5.**
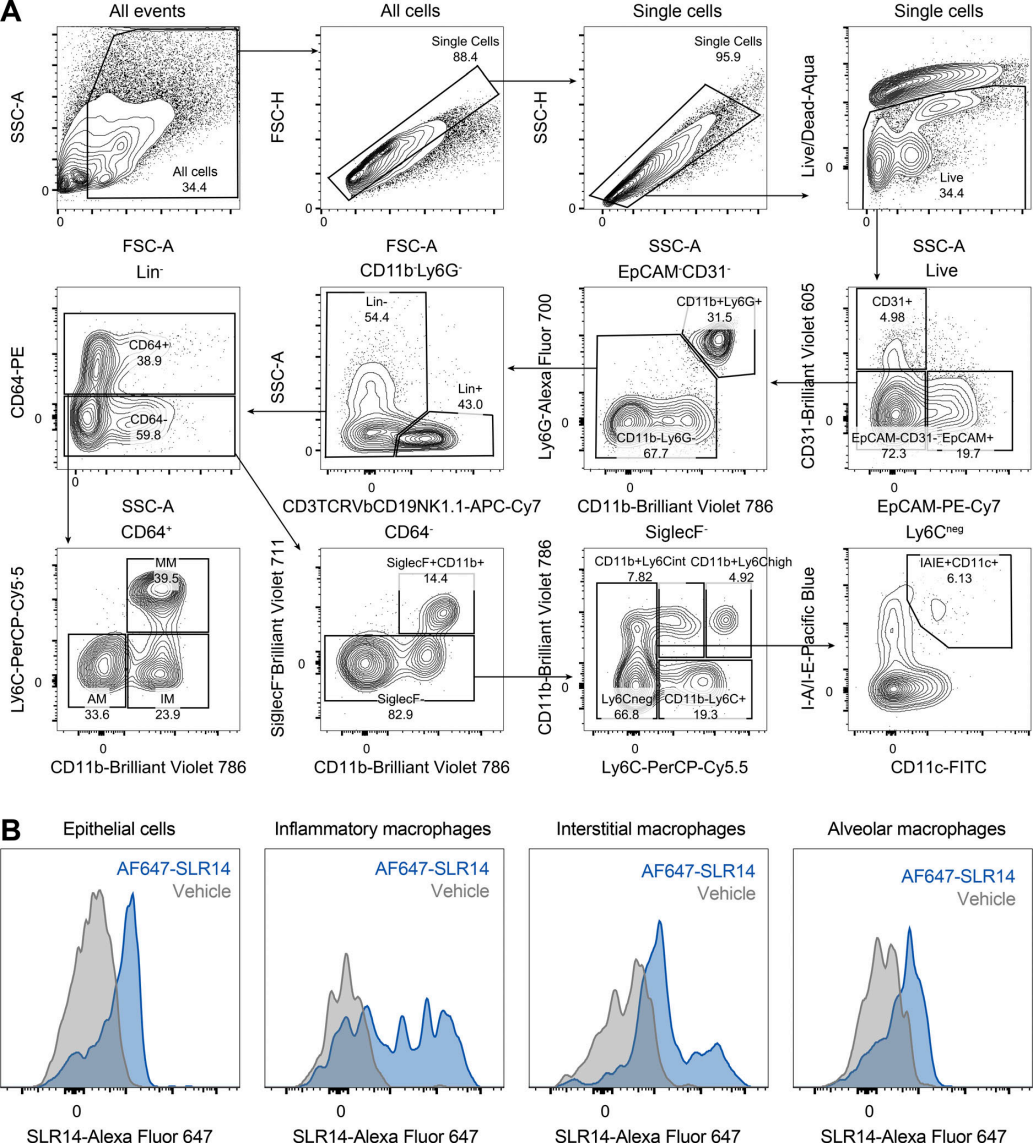
**Gating strategies for identification of various cellular subsets using flow cytometry.** **(A)** Gating strategies for identification of various immune and nonimmune cell populations in the lung were used to generate Fig. 1, I and J and Fig. S3, B–E.** (B)** Histogram examples of SLR14 uptake by epithelial cells and different macrophage subsets. Lung tissues from vehicle-treated mice were included as negative controls.

Mouse lung tissues were collected at experimental end point, digested with 1 mg/ml collagenase A (Roche) and 30 µg/ml DNase I (Sigma-Aldrich) in complete RPMI-1640 media for 30 min at 37°C, and mechanically minced. Digested tissues were then passed through a 70-µm strainer (Thermo Fisher Scientific) to single-cell suspension and treated with ACK Lysing Buffer (Thermo Fisher Scientific) to remove red blood cells. Cells were resuspended in Live/Dead Fixable Aqua (Thermo Fisher Scientific) for 20 min at 4°C. Following a wash, cells were blocked with anti-mouse CD16/32 antibodies (BioXCell) for 30 min at 4°C. Cocktails of staining antibodies were added directly to this mixture for 30 min at 4°C. Prior to analysis, mouse cells were washed and resuspended in 100 µl 4% PFA for 30–45 min at 4°C. Following this incubation, cells were washed and prepared for analysis on an Attune NXT (Thermo Fisher Scientific). Data were analyzed using FlowJo software version 10.6 software (Tree Star). The specific sets of markers used to identify each subset of cells are summarized in Fig. S5.

### SARS-CoV-2–specific antibody ELISA measurement

SARS-CoV-2–specific antibodies were measured as previously described (Israelow et al., 2021). In brief, sera were treated with 0.5% Triton X-100 and 0.5 mg/ml RNase A to inactivate potentially infectious viruses. Recombinant SARS-CoV-2 S1 protein (S1N-C52H3; ACRO Biosystems) was used to coat 96-well MaxiSorp plates (Thermo Fisher Scientific) overnight. The coating buffer was removed, and plates were treated with blocking solution followed by incubation with diluted serum. Plates were washed with PBS-T and HRP anti-mouse IgG antibody were added to each well. After incubation plates were washed with PBS-T and developed with TMB Substrate Reagent Set (BD Biosciences 555214). The reaction was stopped by 2 N sulfuric acid. Plates were then read at a wavelength of 450 nm and 570 nm.

### Determination of IFN-I and IFN-III concentration

Concentration of IFN-I in BAL fluid was determined by ELISA (42120 and 42400; PBL Assay Science) following the manufacturer’s instructions. IFN-α–precoated plates were incubated for 24 h with diluted or undiluted samples and target antibody, followed by a wash with PBS-T. Similarly, IFN-β–precoated plates were incubated with undiluted samples for 1 h and washed with PBS-T. Detection antibody was added and incubated for 1 h, followed by a second wash. Both plates were then treated with HRP solution and washed before addition of TMB substrate solution. The reaction was left to develop for 10 and 15 min, respectively, and stopped with 2 N sulfuric acid. Absorbance was recorded at 450 nm, and background noise was subtracted from the negative control in the experiment. Concentration of IFN-λ in BAL fluid was determined by ELISA (DY1789B; R&D Systems) according to manufacturer’s instructions. 96-well MaxiSorp plates (Thermo Fisher Scientific) were coated overnight with 1 µg/ml coating antibody. After a wash with PBS-T, the plates were blocked with 1% BSA in PBS for 1 h and subsequently incubated for 2 h with diluted or undiluted samples. The plate was washed and incubated for 2 h with detection antibody followed by a third wash. Streptavidin-HRP treatment was performed for 20 min and then washed out before the addition of TMB substrate solution. The reaction was left to develop for 8 min and stopped with 2 N sulfuric acid. Absorbance was recorded at 450 nm, and background noise was subtracted from the negative control in the experiment.

### Adoptive transfer of sera

WT AAV-hACE2 mice were infected with SARS-CoV-2 as indicated above. At 14 DPI, animals were euthanized for blood collection. Blood was allowed to coagulate at room temperature for 30 min and then was centrifuged at 3,900 rpm for 20 min at 4°C. Serum was collected, and anesthetized mice (30% vol/vol isoflurane diluted in propylene glycol) were injected with 200 µl serum with a 32-G 8-mm syringe via the retro-orbital route.

### Statistical analysis

Data were analyzed by log-rank Mantel–Cox test or one-way ANOVA followed by Tukey correction. All statistical tests were calculated using GraphPad Prism. A P value of <0.05 was considered statistically significant.

### Online supplemental material

Fig. S1 illustrates additional histological analyses of lung sections from vehicle- or SLR14-treated mice following SARS-CoV-2 infection. Fig. S2 demonstrates that SLR14 treatment does not induce significant induction of IFN-III response and that SLR14-mediated antiviral effects and its dependency on intact IFN-I signaling can also be seen in the AAV-hACE2 mouse model. Fig. S3 shows that i.v.-injected SLR14 can be taken up by various cell types in the lung, in particular epithelial cells and macrophages. Fig. S4 depicts weight loss of individual mice treated with vehicle or SLR14 following infection with different SARS-CoV-2 variants and partial SLR14 resistance by the Alpha variant. Fig. S5 shows the gating strategy for flow cytometry experiments conducted in this study. Table S1 lists changes in amino acids that were identified by sequencing in clinical isolates of SARS-CoV-2 compared with the reference genome.

## Supplementary Material

Table S1 lists amino acid changes identified in SARS-CoV-2 resequenced after virus isolation as compared to the reference genome (GenBank accession no. MN908947).
