# Supplementary material for: A stem-loop RNA RIG-I agonist protects against acute and chronic SARS-CoV-2 infection in mice
Source: J Exp Med. 2021 Nov 10;219(1):e20211818. doi: 10.1084/jem.20211818 (PMC8590200; doi:10.1084/jem.20211818)
Supplement: Table S1 — lists amino acid changes identified in SARS-CoV-2 resequenced after virus isolation as compared to the reference genome (GenBank accession no. MN908947). [file JEM_20211818_TableS1.docx]

Table S1. Amino acid changes identified in SARS-CoV-2 resequenced after virus isolation as compared to the reference genome (GenBank accession no. MN908947)

|  | B.1.1.7 (GenBank accession no. MZ202178) | B.1.351 (GenBank accession no. MZ202314) | P.1 (GenBank accession no. MZ202306) | B.1.526 (GenBank accession no. MZ201303) | B.1.617.2 (GenBank accession no. MZ468047) |
| --- | --- | --- | --- | --- | --- |
| E |  | P71L |  |  | V62F |
| M |  |  |  |  | I82T |
| N | M1X | T205I | P80R | M1X | D63G |
|  | D3L |  | R203K | P199L | R203M |
|  | R203K |  | G204R | M234I | D377Y |
|  | G204R |  |  |  |  |
|  | S235F |  |  |  |  |
| ORF1a | T1001I | T265I | S1188L | T265I | P309L |
|  | P1213L | K1655N | K1795Q | T2977I | A405V |
|  | A1708D | K3353R | G2941S | L3201P | P1640L |
|  | I2230T | S3675- | S3675- | S3675- | A3209V |
|  | M2259I | G3676- | G3676- | G3676- | V3718A |
|  | S3675- | F3677- | F3677- | F3677- |  |
|  | G3676- |  |  |  |  |
|  | F3677- |  |  |  |  |
| ORF1b | P218L | P314L | P314L | P314L | P314L |
|  | P314L |  | A1219S | Q1011H | G662S |
|  | A1432V |  | E1264D | R1078C | P1000L |
|  |  |  |  |  | P1570L |
| ORF3a |  | Q57H | Q57H | P42L | S26L |
|  |  | W131L | S253P | Q57H |  |
|  |  | S171L |  |  |  |
| ORF7a |  | V93F |  |  | V82A |
|  |  |  |  |  | L116F |
|  |  |  |  |  | T120I |
| ORF8 | Q27* | R115L | E92K | T11I | D119- |
|  | R52I |  |  |  | F120- |
|  | K68* |  |  |  |  |
|  | Y73C |  |  |  |  |
| ORF9b |  |  | Q77E |  | T60A |
| S | H69- | L18F | L18F | L5F | T19R |
|  | V70- | D80A | T20N | T95I | E156- |
|  | Y144- | D215G | P26S | D253G | F157- |
|  | N501Y | L242H | D138Y | E484K | R158G |
|  | A570D | K417N | R190S | D614G | L452R |
|  | D614G | E484K | K417T | A701V | T478K |
|  | P681H | N501Y | E484K |  | D614G |
|  | T716I | D614G | N501Y |  | P681R |
|  | S982A | Q677H | D614G |  | D950N |
|  | D1118H | R682W | H655Y |  | L1141W |
|  |  | A701V | T1027I |  |  |
|  |  | A243- | V1176F |  |  |
|  |  | L244- |  |  |  |
|  |  | H245- |  |  |  |

Listed are amino acid substitutions and deletions for each of the genes. Letters indicate amino acids, numbers indicate amino acid positions, asterisks indicate stop codon mutations, and dashes indicate deletions. E, envelope protein, N, nucleocapsid protein, ORF, open reading frame; S, spike protein.
